# Automated quality control of small animal MR neuroimaging data

**DOI:** 10.1162/imag_a_00317

**Published:** 2024-10-17

**Authors:** Aref Kalantari, Mehrab Shahbazi, Marc Schneider, Adam C. Raikes, Victor Vera Frazão, Avnish Bhattrai, Lorenzo Carnevale, Yujian Diao, Bart A. A. Franx, Francesco Gammaraccio, Lisa-Marie Goncalves, Susan Lee, Esther M. van Leeuwen, Annika Michalek, Susanne Mueller, Alejandro Rivera Olvera, Daniel Padro, Mohamed Kotb Selim, Annette van der Toorn, Federico Varriano, Roël Vrooman, Patricia Wenk, H. Elliott Albers, Philipp Boehm-Sturm, Eike Budinger, Santiago Canals, Silvia De Santis, Roberta Diaz Brinton, Rick M. Dijkhuizen, Elisenda Eixarch, Gianluigi Forloni, Joanes Grandjean, Khan Hekmatyar, Russell E. Jacobs, Ileana Jelescu, Nyoman D. Kurniawan, Giuseppe Lembo, Dario Livio Longo, Naomi S. Sta Maria, Edoardo Micotti, Emma Muñoz-Moreno, Pedro Ramos-Cabrer, Wilfried Reichardt, Guadalupe Soria, Giovanna D. Ielacqua, Markus Aswendt

**Affiliations:** University of Cologne, Faculty of Medicine and University Hospital Cologne, Department of Neurology, Cologne, Germany; Hamedan University of Technology, Faculty of Medical Engineering, Hamedan, Iran; Center for Innovation in Brain Science, University of Arizona, Tucson, AZ, USA; IRCCS INM Neuromed, Department of AngioCardioNeurology and Translational Medicine, Pozzilli, Italy; Department of Radiology, Lausanne University Hospital (CHUV), Lausanne, Switzerland; CIBM Center for Biomedical Imaging, Lausanne, Switzerland; Translational Neuroimaging group, Center for Image Sciences, University Medical Center Utrecht, Utrecht, The Netherlands; Institute of Biostructures and Bioimaging (IBB), National Research Council of Italy (CNR), Turin, Italy; Leibniz Institute for Neurobiology (LIN), Combinatorial Neuroimaging Core Facility (CNI), Magdeburg, Germany; Center for Behavioral Neuroscience, Neuroscience Institute, Advanced Translational Imaging Facility, Georgia State University, Atlanta, Georgia, USA; Charité 3R | Replace, Reduce, Refine, Charité-Universitätsmedizin Berlin, corporate member of Freie Universität Berlin and Humboldt-Universität zu Berlin, Berlin, Germany; Department of Experimental Neurology and Center for Stroke Research Berlin, Charité-Universitätsmedizin Berlin, Berlin, Germany; NeuroCure Cluster of Excellence and Charité Core Facility 7T Experimental MRIs, Charité-Universitätsmedizin Berlin, Berlin, Germany; Donders Institute for Brain, Cognition, and Behaviour, Radboud University Medical Centre, Nijmegen, The Netherlands; Center for Cooperative Research in Biomaterials (CIC biomaGUNE), Basque Research and Technology Alliance (BRTA), Donostia-San Sebastián, Spain; Instituto de Neurociencias, CSIC/UMH, San Juan de Alicante, 03550 Alicante, Spain; BCNatal Fetal Medicine Research Center (Hospital Clínic and Hospital Sant Joan de Déu), Universitat de Barcelona; Institut d’Investigacions Biomèdiques August Pi i Sunyer (IDIBAPS); and Centre for Biomedical Research on Rare Diseases (CIBER-ER), Spain; Istituto di Ricerche Farmacologiche Mario Negri IRCCS, Department of Neuroscience, Milan, Italy; Department of Medical Imaging, Radboud University Medical Center, Nijmegen, The Netherlands; Department of Physiology and Neuroscience, Keck School of Medicine, University of Southern California, Los Angeles, CA, USA; Centre for Advanced Imaging, Australian Institute for Bioengineering and Nanotechnology, The University of Queensland, St. Lucia, Australia; Sapienza University of Rome, Department of Molecular Medicine, Rome, Italy; Molecular Imaging Center, Department of Molecular Biotechnology and Health Sciences, University of Turin, Turin, Italy; MRI Core Facility, Institut d’Investigacions Biomèdiques August Pi i Sunyer (IDIBAPS), Barcelona, Spain; Ikerbasque, Basque Foundation for Science, Bilbao, Spain; Division of Medical Physics, Department of Diagnostic and Interventional Radiology, University Medical Center Freiburg, Faculty of Medicine, University of Freiburg, Freiburg, Germany; CIBER de Bioingeniería, Biomateriales y Nanomedicina, Instituto de Salud Carlos III; Laboratory of Surgical and Experimental Neuroanatomy, Faculty of Medicine and Health Sciences, Institute of Neurosciences, University of Barcelona, Barcelona, Spain; Preclinical Research Center (PRC), Max-Delbrück Center for Molecular Medicine in the Helmholtz Association, Berlin, Germany; Center for Behavioral Brain Sciences, Magdeburg, Germany

**Keywords:** standardization, reproducibility, machine learning, motion detection, image artifacts, majority voting

## Abstract

Magnetic resonance imaging (MRI) is a valuable tool for studying brain structure and function in animal and clinical studies. With the growth of public MRI repositories, access to data has finally become easier. However, filtering large datasets for potential poor-quality outliers can be a challenge. We present AIDAqc, a machine-learning-assisted automated Python-based command-line tool for small animal MRI quality assessment. Quality control features include signal-to-noise ratio (SNR), temporal SNR, and motion. All features are automatically calculated and no regions of interest are needed. Automated outlier detection for a given dataset combines the interquartile range and the machine-learning methods one-class support vector machine, isolation forest, local outlier factor, and elliptic envelope. To evaluate the reliability of individual quality control metrics, a simulation of noise (Gaussian, salt and pepper, speckle) and motion was performed. In outlier detection, single scans with induced artifacts were successfully identified by AIDAqc. AIDAqc was challenged in a large heterogeneous dataset collected from 19 international laboratories, including data from mice, rats, rabbits, hamsters, and gerbils, obtained with different hardware and at different field strengths. The results show that the manual inter-rater agreement (mean Fleiss Kappa score 0.17) is low when identifying poor-quality data. A direct comparison of AIDAqc results, therefore, showed only low-to-moderate concordance. In a manual post hoc validation of AIDAqc output, precision was high (>70%). The outlier data can have a significant impact on further postprocessing, as shown in representative functional and structural connectivity analysis. In summary, this pipeline optimized for small animal MRI provides researchers with a valuable tool to efficiently and effectively assess the quality of their MRI data, which is essential for improved reliability and reproducibility.

## 1 Introduction

Quality control (QC) is an integral part of overcoming reproducibility issues and lack of transparency toward open data sharing for magnetic resonance imaging (MRI) (Niso et al., 2022). Numerous software developments are available for human MRI, for example, MRIQC (Esteban et al., 2017), pyfMRIqc (Williams et al., 2023), VisualQC (Raamana, 2018), and FreeSurfer (Backhausen et al., 2016) as well as standardized protocols, for example, in the Human Connectome Project (Marcus et al., 2013), ENIGMA (Thompson et al., 2020), and INDI initiative (Zarrar et al., 2015). In contrast, the focus of previous small animal approaches was mainly on the use of standardized phantoms but not software (Mannheim et al., 2018;Osborne et al., 2017). A recent survey highlighted the need for more efficient animal MRI QC, as the majority of users (63%) do not follow any QC protocols and 19% declare that no regular maintenance for quality assurance (QA) of the machine is performed (Tavares et al., 2023). This is in stark contrast to industry and clinical guidelines, for example, the AAPM acceptance testing and QA procedures for MRI facilities (Jackson et al., 2010) or the ACR MRI QC manual (Price et al., 2015), which have been shown to improve further MRI analysis (Bedford et al., 2023;Gilmore et al., 2021).

Both QA and QC standards aim to facilitate stable, high-quality MRI data. In addition to the desired homogeneous image quality in the in-house laboratory, QC/QA procedures are necessary for longitudinal imaging sessions between different laboratories (Sreedher et al., 2021). MR image quality is mainly determined by noise and image artifacts. Noise arises from multiple sources including intrinsic scanner and thermal noise as well as physiological noise, which originates from motion, cardiac cycle, and respiration (Bianciardi et al., 2009). Typical image artifacts include occasional noise, differences in susceptibility, that is, differences in the magnetic field, mostly at the border between different tissue compartments (Budrys et al., 2018), and ghosting, that is, a blurry copy of the sample that appears in the field-of-view displaced from its true location. Ghosting can be induced by periodic movements, including involuntary movements, cardiac and respiratory motion, vessel pulsation, and blood and CSF flow (*discrete ghosts*) (Zaitsev et al., 2015) or hardware- and software-related issues, such as improper shimming, strong heating of the gradients, and induction of eddy currents (*Nyquist ghosts*) (Chen & Wyrwicz, 2004). In small animal imaging, movement is restricted by anesthesia and animal fixation in the MRI bed, which can be combined with muscle relaxants and intubation to achieve a stable respiratory and cardiac cycle (Grandjean et al., 2014). Nevertheless, physiological factors and movement of parts of the body can induce motion in the image. This leads to an increased level of noise, blurring, and ghosting artifacts (Zaitsev et al., 2015), which are difficult to distinguish and eliminate via postprocessing. Due to the large variety of hardware/software combinations, the most prominent components influencing image quality may vary. For functional MRI (fMRI) in rodents, a previous study has shown that physiological fluctuations, mainly originating from the respiratory cycle, introduce severe artifacts and lead to misinterpretation of corresponding connectivity measures (Kalthoff et al., 2011). Different from faster MRI sequences with easier visual interpretation of motion artifacts, for example, as ghosting or blurring, in dynamic MRI scans, motion artifacts can cause signal changes that may severely confound statistical analysis rendering results unreliable (Havsteen et al., 2017).

The manual identification of poor image quality, which is still the most frequently used method, can only be practically applied to a rather small number of datasets (according to our expertise this relates to <50 datasets) and remains a very subjective method with high interassessor variability (Williams et al., 2023). This is mainly due to unavoidable human error and a missing standard of how to draw and where to place regions of interest (ROI) to quantify signal-to-noise ratio (SNR) or temporal SNR (tSNR). Predefined standardized regions as implemented in the image artifact analysis of the American College of Radiology are only available for phantoms (Etman et al., 2017). As an alternative, machine-learning algorithms for specific quality metrics were tested, for example, to automatically detect motion artifacts (Fantini et al., 2021;Lorch et al., 2017). However, such deep learning approaches require large training datasets, which is difficult to achieve with small animal MRI and in addition would require expert knowledge to generate a ground truth. Automation without the need to train the model would provide a unique opportunity to assess the quality of images for less experienced users or a scenario of data reuse. However, to date, user-independent and large-scale-applicable calculation of image quality metrics in small animal MRI is missing. To address this issue, we developed AIDAqc, a user-friendly command-line tool tailored to the needs of small animal MR neuroimaging, while maintaining high flexibility with respect to animal model, MR hardware, and sequences. The tool generates a detailed summary of the dataset parameters and performs automatic calculations of image quality metrics, which are assessed using a majority-voting approach to simplify and objectify the categorization of image quality. Here, we describe the AIDAqc pipeline, present the validation using a large heterogeneous dataset collected from 19 international MRI laboratories, and discuss the interpretation of quality metrics and their influence on further analysis.

## 2 Methods

### 2.1 Python-based Workflow for automated QC

AIDAqc was developed as part of the AIDA (Atlas-based Imaging Data Analysis Pipeline) software family (https://github.com/aswendt-lab) with the suffix*qc*relating to*quality control*. The automated pipeline was tested in Python 3.12.0 using the libraries*Numpy*,*Pandas*,*Matplotlib*,*Sklearn*,*Nibabel*, and*Alive_progress*. The installation was tested successfully using a custom Anaconda software environment (conda 23.10.0), on Windows 10 & 11, macOS 12.6.8, and Linux Ubuntu 22.04. Detailed instructions on how to install and use AIDAqc can be found online (https://github.com/Aswendt-Lab/AIDAqc).

**Fig. 1.**
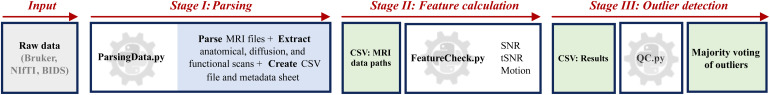
Schematic overview of the AIDAqc pipeline. Starting with input data formats, three consecutive stages of automatic parsing, feature calculation, and multiple outlier detection techniques combined to a major vote for the outlier status. Note: naming convention—anatomical (all T1- or T2-weighted scans), diffusion (all diffusion-weighted, DWI, or diffusion tensor imaging, DTI, scans), and functional (fMRI, and rs-fMRI scans).

The following subsections explain each stage of the pipeline in detail (Fig. 1): (1). Parsing Input Data, (2). Feature Calculation, and (3).Outlier Detection.

#### 2.1.1 Stage I: Parsing input data

To increase the flexibility of the pipeline, the Neuroimaging Informatics Technology Initiative (NIfTI) data format, Brain Imaging Data Structure (BIDS) data folder structure, as well as the raw Bruker (Bruker, BioSpin, Ettlingen, Germany) data format (i.e., the complete Bruker study folder including image and metadata from one subject and time point) can be used as input. Note: the image data need to be oriented as coronal brain sections. In the initial parsing stage, the input folder is automatically and recursively searched for these MRI files. As a result, the file paths are validated to contain a unique file and sorted into anatomical (T1- or T2-weighted), diffusion (diffusion-weighted, diffusion tensor imaging, etc.), and functional (fMRI and rs-fMRI) scans based on filenames and related metadata. Other scans, for example, localizers, and files with missing necessary metadata are excluded from further processing.

#### 2.1.2 Stage II: Feature calculations

Automatically calculated features include (t)SNR, motion, and ghosting. At this stage, the quantitative measures are automatically obtained from the input data, that is, no user input is required. Specific measures, for example, tSNR, will be calculated only for fMRI.

##### 2.1.2.1 Snr

The SNR is the most rudimentary but also versatile quantitative measure of MR image quality for most sequences. However, the calculation can get complex due to the manual definition of regions of interest (ROIs), that is, at least one ROI inside the*sample*and one ROI outside the sample representing the*noise area*. Here, two approaches were implemented: the “standard” method, which automatically defines ROIs based on the center of intensity (COI) of the image, and the Chang method (Chang et al., 2005), which is independent of ROI selection. For both methods, the SNR is reported in decibel (dB).

###### 2.1.2.1.1 Standard method

**Fig. 2.**
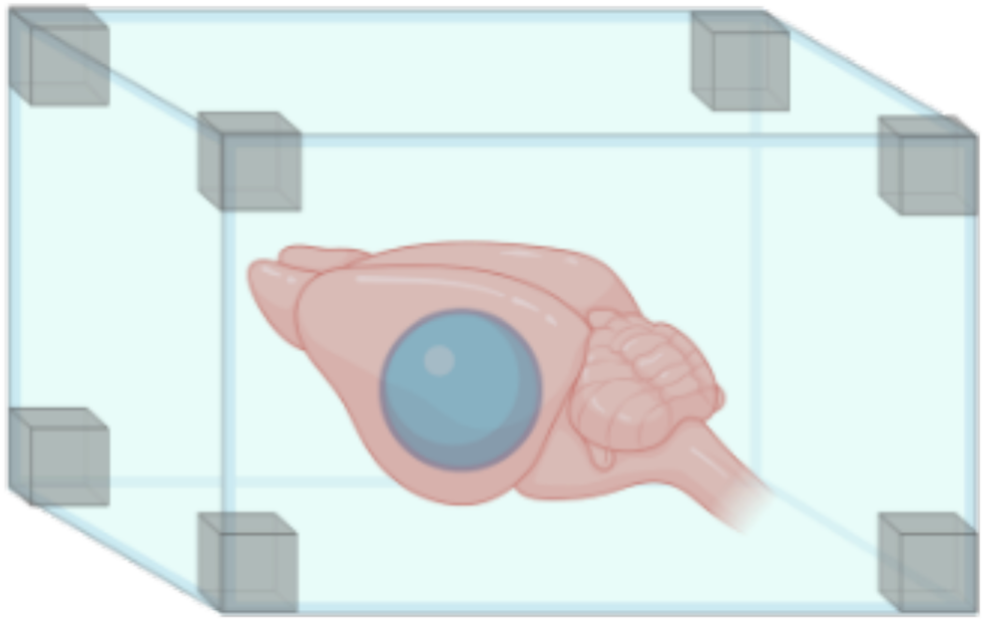
SNR calculation without manually placed regions of interest (ROI). Graphical illustration of automated ROI selection to distinguish signal (blue sphere) from noise (gray cuboids), which are used to calculate the SNR.

SNR is calculated by dividing the mean signal intensity by the standard deviation of the noise (Henkelman, 1985;Kaufman et al., 1989). ROIs are placed automatically in a three-step procedure (Fig. 2).

1. **Center of intensity (COI):**To robustly determine the center of the object in the image, the coordinates of the COI are extracted as a starting point for step 2.
2. **COI sphere:**A sphere with the COI as its center is used to create a mask for averaging all the voxels within this sphere as a reference value for the “true signal.” To ensure that the sphere does not extend beyond the signal volume of interest, a relative value for the sphere radius is set based on the image dimensions.
3. **Cuboids:**In all eight corners of the image volume with a size relative to the dimensions of the volume, cuboid ROIs are created. All voxels in these cuboids are averaged and used as a reference for the*noise*.

If*I*represents the three-dimensional image, then*S*and*C*are subsets of*I*representing the sphere and the cuboid space, respectively (Eq. 1a).*S(x,y,z)*and*C(x,y,z)*are image functions representing a specific voxel in*I*, defined by the coordinate*x,y,z*in the three-dimensional image space*I*. Upper limits of the subsets*S*and*C*are shown as*($i_{s},\;j_{s},\;k_{s}$)*and*($i_{c},\;j_{c},\;k_{c}$)*, respectively (Eq. 1a). The SNR is calculated by dividing the mean intensity of the sphere by the standard deviation of the cuboids with*N*being the number of voxels in the image subset of the image*I*(Eq. 1b). The SNR value is commonly reported in decibel, dB (Eq. 1c). In the case of 4D diffusion images, the first b0 image volume is chosen to represent the three-dimensional image*I*.

(x, y, z), C(x, y, z)⊂I(x,y, z) (1a)

$$\mu_{S}=\frac{\sum_{x=1\;}^{i_{s}}\sum_{y=1\;}^{j_{s}}\sum_{z=1}^{k_{s}}\;S\left(x,y,z\right)}{N_{s}},\sigma_{\text{c}}=\sqrt{\frac{\sum_{x=1\;}^{i}\sum_{y=1\;}^{j}\text{​}\sum_{z=1}^{k}{\left(C\left(x,y,z\right)-\mu_{c}\right)}^{2}}{{\text{N}}_{\text{c}}}}$$ (1b)

$$SNR_{standard}=\frac{\mu_{s}}{\sigma_{c}}=20\cdot \log\left(\frac{\mu_{s}}{\sigma_{c}}\right)\text{ dB}.$$ (1c)

###### 2.1.2.1.2 Chang method

This approach uses a histogram analysis of the image signal intensity to calculate the distribution of noise (Chang et al., 2005). The strength of the method lies in the fact that it can be used for developing a processing workflow for large-scale MR data without the need for manual region selection. Here, this method is used to acquire a value for SNR based onEquation 2with*E (z)*as the SNR calculated for each slice (z) with the Chang function (see details of the function inChang et al. (2005)).*N*is the number of voxels in the image subset of the image*I*. In the case of 4D diffusion images, the Chang method (different to the standard method) creates an average SNR of all diffusion directions except the b0 image.

$$\mu_{slice}(z)=\frac{\sum_{x=1\;}^{N}\sum_{y=1}^{N}S\left(x,y,z\right)}{N_{s}},\sigma_{\text{chang}}(z)=\text{Chang}\left(I(x,y,z)\right)$$ (2a)

$$E(z)=\frac{\mu_{\text{slice}}(z)}{\sigma_{\text{chang}}(z)}=20\cdot \log\left(\frac{\mu_{\text{slice}}(z)}{\sigma_{\text{chang}}(z)}\right)\text{ dB}$$ (2b)

$${\text{SNR}}_{\text{chang, Final}}=\frac{\sum_{z=1}^{Slices}E(z)}{N}.$$ (2c)

##### 2.1.2.2 tsnr

The temporal signal-to-noise ratio (tSNR) is defined for sequences acquired over time (Welvaert & Rosseel, 2013). Therefore, there is an additional fourth dimension in a three-dimensional volume, here represented with the image functionI(x, y, z, t)inEquation 3. For this case, the center of intensity (COI) of the averaged volume over time is calculated and the sphere with the COI as its center is defined. The sphere image function as a subset of S is represented withS(x, y, z, t).${\text{N}}_{\text{s}}$is the number of voxels in the sphere. From a practical point of view, to calculate one quantitative value for the tSNR, first, a tSNR map is calculated (Eq. 3c) and the average of the tSNR over all voxels in the sphere is the final tSNR value.

S(x, y, z, t)⊂I(x, y, z, t) (3a)

$$\mu_{t}\left(x,\;y,\;z\right)=\frac{\sum_{t=1\;}^{T}S\left(x,\;y,\;z,\;t\right)}{N_{s}},\sigma_{t}\left(x,y,z\right)=\sqrt{\frac{\sum_{t=1}^{T}{\left(S\left(x,y,z,t\right)-\mu_{t}\left(x,y,z\right)\right)}^{2}}{N_{s}}}$$ (3b)

$$\text{tSNR}\left(\text{x},\;\text{y},\;\text{z}\right)=\frac{\mu_{\text{t}}\left(\text{x},\;\text{y},\;\text{z}\right)}{\sigma_{\text{t}}\left(\text{x},\;\text{y},\;\text{z}\right)}=20\log\left(\frac{\mu_{\text{t}}\left(\text{x},\;\text{y},\;\text{z}\right)}{\sigma_{\text{t}}\left(\text{x},\;\text{y},\;\text{z}\right)}\right)\text{ dB}$$ (3c)

$${\text{tSNR}}_{\text{Final}}=\sum_{\text{x}=1\;}^{{\text{i}}_{\text{s}}}\sum_{\text{y}=1}^{{\text{j}}_{\text{s}}}\;\sum_{\text{z}=1}^{{\text{k}}_{\text{s}}}\text{tSNR}\left(\text{x},\;\text{y},\;\text{z}\right).$$ (3d)

##### 2.1.2.3 Ghosting and motion

**Fig. 3.**
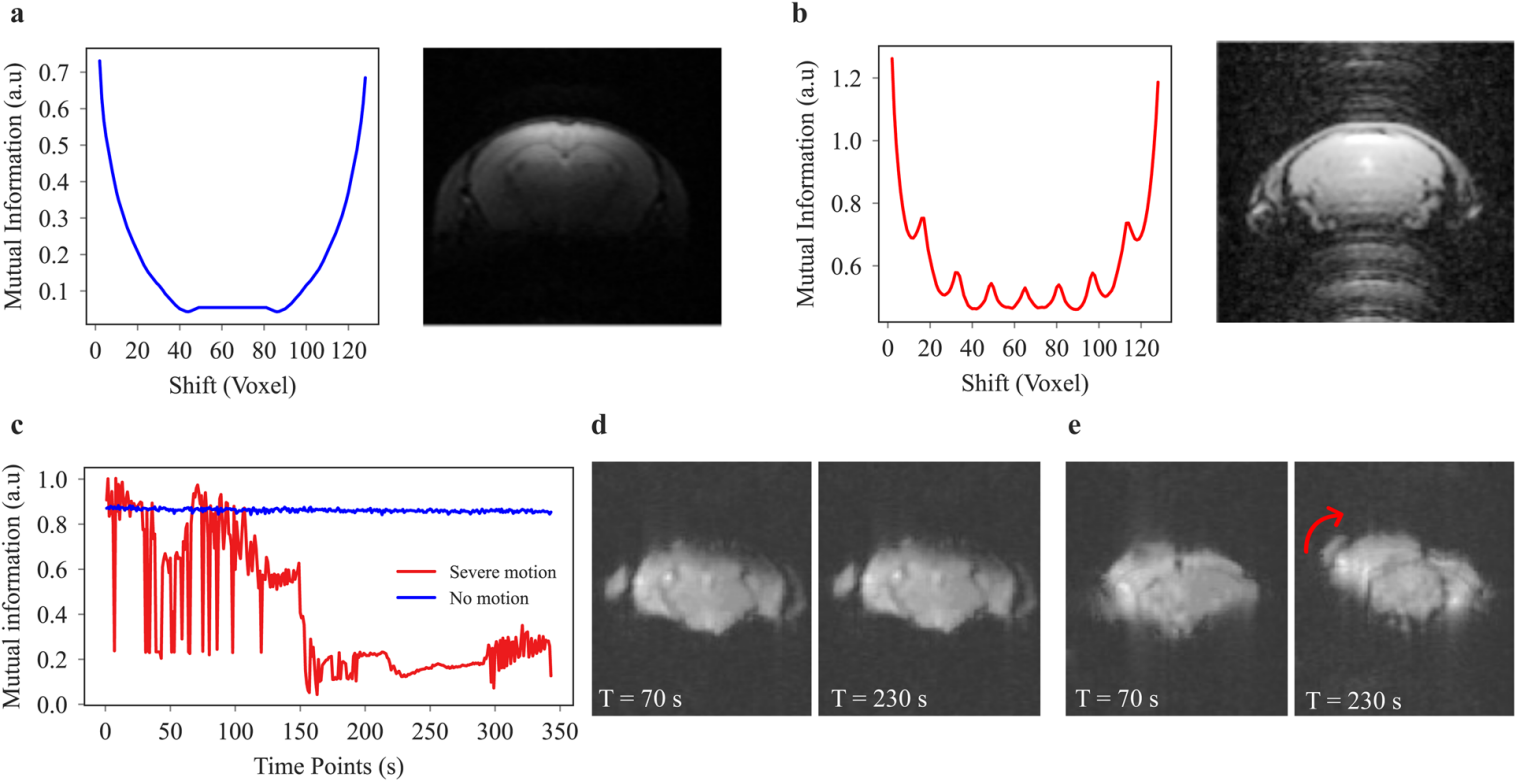
Mutual information (MI) to detect ghosting artifacts and motion. (a) Image without ghosting: smooth and bowl-shaped MI curve indicating the whole cycle of image shifting. An initial decrease due to an increasing mismatch between the reference image and the shifted versions is followed by an increase to its initial state when the shifted image arrives at the reference. (b) Complementary MI curve for an image with ghosting. MI peaks at various shifts throughout the cycle. (c) Representative MI was plotted for consecutive time points of two rs-fMRI scans, one with strong motion artifacts and the other with no motion artifacts. (d) There is near to no motion visually detectable in the receptive images of time = 70 versus 230 s. (e) The motion can be detected visually in the receptive images of time = 70 versus 230 s. A mixture of translational and rotational motions shifts the image approximately 20 voxels.

Mutual information (MI) was used as a metric for the identification of MRI ghosting artifacts (Fig. 3a, b). It is known that one of the sources of ghosting artifacts is the sensitivity of echo-planar imaging (EPI) scans to magnetic field inhomogeneities and an additive consequence of hardware-originated artifacts. The*Nyquist ghost*is one of the artifacts happening with EPI sequences where the ghost is located half of the field of view (FOV) away from the image (Buonocore & Gao, 1997;Reeder et al., 1997;Yang et al., 1996). Additionally, there are*discrete ghosts*, which occur in images where the periodic motion of the subject is present, like respiratory or cardiac motions. These regular motions often spread across the whole image in the form of ghosts emerging from the principles of the Fourier transform (Axel et al., 1986;Storey et al., 2002;Wood & Henkelman, 1985).

In practice, irrespective of the type of the ghost for a 3D image volume, the middle slice is automatically extracted. For 4D image data, that is, diffusion and functional scans, the average across the fourth dimension is used to create a representative 3D image. In the next step, the MI is calculated between the selected image and the shifted image by*n*voxels, with*n*ranging from 1 to N, with N being the size of the image in x or y directions. According to the MI theorem, the MI value decreases as the shifts increase because the pair of images fits less well. Considering a “ghost” has happened, along one of these shifted image versions, the main target of the image (in our case the brain) will match its ghost in the shifted image. This process will generate peaks in the MI as the shifts increase, which are automatically detected. If a peak exceeds a defined threshold, there is a high probability that it is due to a ghost.

Mutual information (MI) is also a sensitive measure of detecting translational motion between images (Godenschweger et al., 2016) and was here used to compare brain structures along the time dimension (Fig. 3c). To obtain a quantitative value for the severity of motion in the image, the Mutual Information (MI) was calculated between a reference image and subsequent images for the time dimension in functional scans and the direction dimension in diffusion scans. The choice of reference image depends on the number of repetitions in the fourth dimension. For smaller datasets with, for example, 10 repetitions, the 1st repetition (TR or b = 0, depending on the acquisition type) is taken as the reference. However, for larger datasets with more repetitions, the 10th repetition is used as the reference to ensure the signal has reached a steady state and is free from initial transient effects. This practice helps to provide a more reliable measure of motion by avoiding the instability often present in the first few repetitions. In the case of no or small amounts of motion (Fig. 3d), the MI is close to 1 and relatively stable over different time points or directions. In the case of severe motion (Fig. 3e), there is a characteristic drop in the MI (Fig. 3c). Motion severity as a single quantitative value for each scan reflects the standard deviation of the MI vector. To reduce the processing time for 4D image volumes, MI is only calculated for the slice with the highest average intensity over time.

#### 2.1.3 Stage III: Outlier detection

After the extraction of quality features, AIDAqc applies one univariate and four multivariate outlier detection methods using a selection of statistical and machine-learning (ML) algorithms:*interquartile range (IQR)*,*one-class support vector machine (ocSVM)*,*isolation forest (IF)*,*local outlier factor (LOF)*, and*elliptic envelope (EE)*.

This approach provides a diverse range of perspectives on the potential outlier status and overcomes the biased approach of only applying one algorithm, which is not suited to detect outliers from a broad range of datasets. The IQR identifies outliers by measuring data spread and is particularly effective for datasets with non-normal distributions (Vinutha et al., 2018). The ocSVM specializes in anomaly detection by defining hyperplanes, making it robust to high-dimensional datasets and capable of capturing complex patterns. The LOF detects anomalies based on local density deviations, offering sensitivity to local variations in data density and robustness to noise, complementing the limitations of ocSVM in handling local anomalies (Budiarto et al., 2019). In scenarios where the ocSVM might struggle with nonlinear separations, Isolation Forest (IF) excels in isolating anomalies by constructing decision trees, providing a computationally efficient alternative (Mohammed et al., 2021). The EE approach offers probabilistic outlier detection by fitting robust covariance estimations, making it suitable for multivariate datasets with elliptical distributions (Ashrafuzzaman et al., 2020).

Outcomes from this multialgorithm approach are aggregated using majority voting on a per-image basis. Each image is assigned a score ranging from 0 (not detected as an outlier in any algorithm) to 5 (reported as an outlier in all algorithms). This enables flexible thresholding on a per-study basis (rather than a singular, binary “outlier” assignment) and minimizes the potential for false positives and negatives in a single-algorithm approach.

#### 2.1.4 Scan reports and automatically created files

**Fig. 4.**
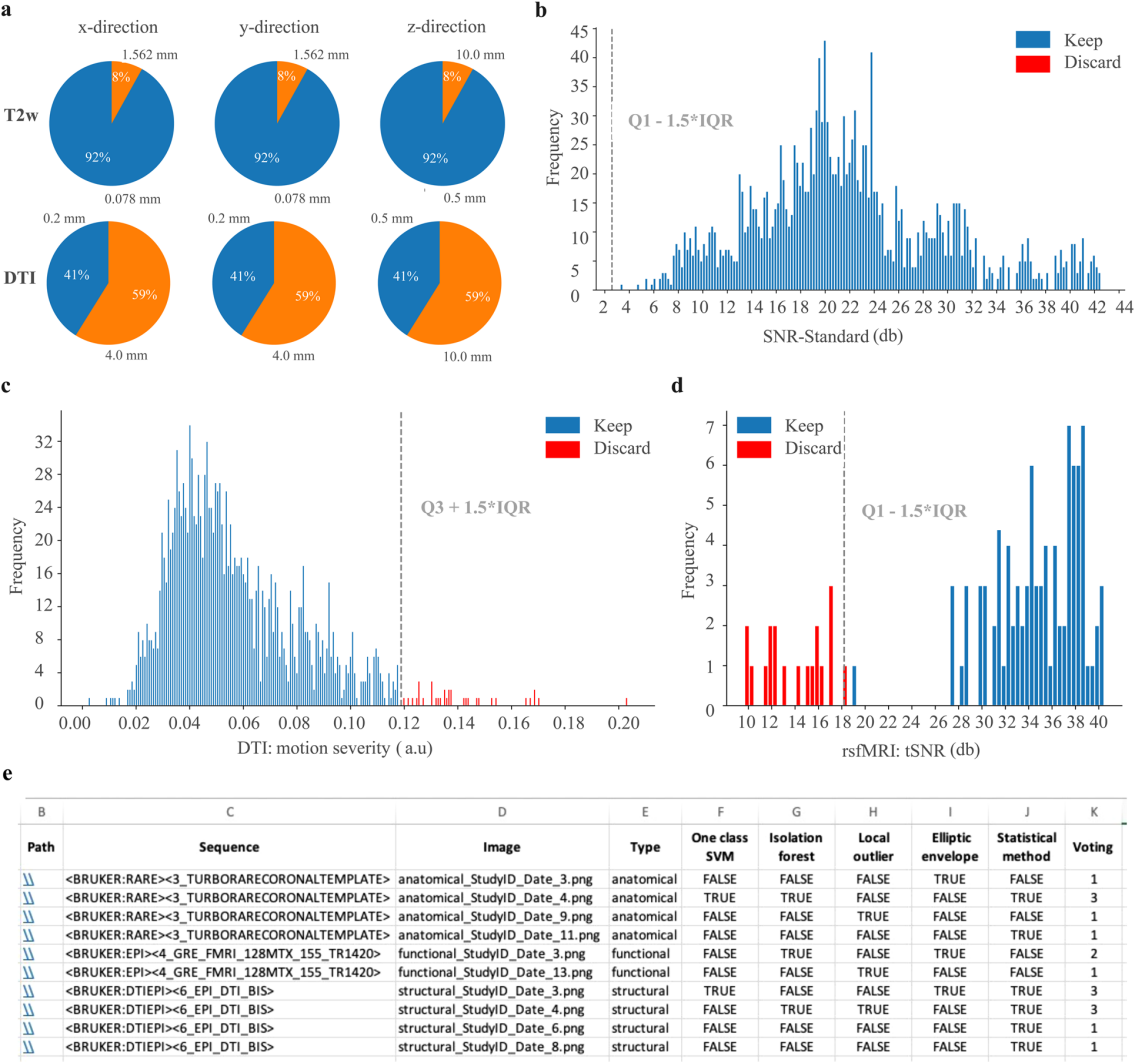
Example of the automatically created outputs as part of the scan report for a representative dataset. Graphs and tables summarizing the (a) spatial resolution (in x-, y-, and z-direction), (b) distribution of anatomical SNR, (c) distribution of motion based on the standard deviation of the mutual information calculated for diffusion scans, (d) distribution of the tSNR for functional scans, and (e) file location, metadata details, and individual outlier votings as listed in the votings.csv file (representative dataset: 94_m_As). Note: in (b–d) red bars indicate outliers based on the statistical definition of quartiles (Q) added by the 1.5-fold interquartile range (IQR). The dashed gray line indicates the predefined outlier threshold. In (e) the file paths were shortened by the placeholder StudyID and date.

AIDAqc provides detailed reports for the user to get an overview of the dataset and to verify the outlier detection in machine-readable CSV files. The reports include information on spatial resolution and are presented in pie charts and distribution plots (Fig. 4). Positive votes that an algorithm has detected an image as an outlier are included in the “*voting.csv*” table, together with the evaluations provided by each outlier approach. This way, the user can identify which algorithm was important to flag the data as an outlier and investigate the reasons further if necessary. Next to the scan report, the single-slice inspection function automatically saves the middle slice images in the respective output folder from each anatomical, diffusion, and functional scan, respectively. The images are named based on a unique scheme, that is, sequence type followed by a number that is incremented for each image and the subject name. Additionally, the image file names are listed in the final feature calculated CSV files.

This information fulfills the purpose to (i) get a first overview of the dataset and to identify scans with different voxel sizes—especially when accidentally a wrong sequence or parameters were used. This feature facilitates quick browsing of images without the need to open each dataset separately in a different program. The output is restricted to the middle slice, which is particularly valuable as it most likely covers larger areas of the subject. While the pipeline is running, users can observe how these images are created one after the other. This allows for quick visual inspection of the subjects. If the orientation of the slice differs in one of these images, the users will promptly recognize that some settings for that subject were different from the usual pattern compared with the others. Furthermore, noisy images are easily discernible at first glance, further enhancing the efficiency of the inspection process. This method can be regarded as a basic but convenient complement to the pipeline. In addition to BIDS datasets, which come with specific metadata and unique file and sequence names, detailed listing as*csv*files is implemented in AIDAqc for Bruker datasets as well. Here, the sequence names, which have been assigned manually by the user, are listed next to the sequence type (Fig. 4eandSupplementary Fig. 9). Further practical instructions on how to interpret AIDAqc results are documented online (https://github.com/Aswendt-Lab/AIDAqc).

### 2.2 Artifact simulation to evaluate the reliability of quality metrics and qutlier detection

#### 2.2.1 Quality metrics

To evaluate the reliability of individual quality control (QC) metrics, a simulation of noise and motion was performed using custom-written code in Python (version 3.11) using the scikit-image library (van der Walt et al., 2014) and Matlab (Matlab Version R2023a, The MathWorks Inc., Natick, USA), respectively. Anatomical and functional scans from the combined datasets 94_m_As and 94c_m_As (Table 1) were used.

**Table 1.** Summary of datasets used for developing AIDAqc.

#	Repository	Datasets	Sequences	Data format	#	Repository	Datasets	Sequences	Data format
**1**	Aswendt	**94_m_As**	T2w, fMRI, DWI	Bruker	**13**	Rivera-Olvera	**117_m_Ri**	T2w, DWI, fMRI	Bruker
**2**	Aswendt	**94c_m_As**	T2w, fMRI, DWI	Bruker	**14**	Sta Maria	**7_m_St**	T2w, DWI	Nifti
**3**	Boehm-Sturm	**7_m_Bo**	T2w, DWI	Bruker	**15**	Selim	**7_m_Se**	T2w, T1w, DWI	Bruker
**4**	Carnevale	**7_m_Ca**	T2/DWI	Bruker	**16**	Selim	**7_r_Se**	T2w, T1w, DWI	Bruker
**5**	Franx	**94_r_Fr**	T2w, DWI, T1w	Nifti	**17**	Soria	**7_r_So**	T2w, fMRI	Nifti
**6**	Hekmatyar	**7_h_He**	T2w, fMRI	Nifti	**18**	Van Leeuwen	**94_m_Va**	T2w, DWI	Nifti
**7**	Kurniawan	**164_m_Ku**	T2w, DWI	Bruker	**19**	Vrooman	**117_m_Vr**	T2w, fMRI	Nifti
**8**	Micotti	**7_m_Mi**	T2w, DWI	Bruker	**20**	Wenk	**94_m_We**	T2w, fMRI, DWI	Bruker
**9**	Muñoz-Moreno	**7_rab_Mu**	T1w, DWI	Nifti	**21**	Wenk/Goncalves	**94_r_We**	T2w, fMRI, DWI	Bruker
**10**	Brinton	**7_m_Br**	T2w, DWI	Nifti	**22**	Wenk/Michalek	**94_g_We**	T2w, fMRI, fMRI, DWI	Bruker
**11**	Ramos-Cabrer	**117_m_Ra**	T2w, DWI, fMRI	Bruker	**23**	Longo	7_m_Lo*	T2w, DWI	Bruker
**12**	Reichardt	94_m_Rei*	T2w	Bruker					

All of the subjects were induced with noise and then AIDAqc was applied on ~60 subjects (30 original, 30 noise induced). This was done to prove the reliability of each metric.

For noise simulation, a constant amount of noise (variance of 0.2) was added to every subject’s anatomical scans. Additionally, a gamma function was applied to reduce the overall brightness of the images, mimicking the shadows present in MR images (gamma value of 0.6). For motion simulation, the spatial image dimensions of the functional scans were manipulated by adding variable translational motion along the time dimension. Three different types of noise were added: Gaussian, salt and pepper (S&P), and speckle noise. Each type of noise serves a specific purpose: Gaussian noise simulates general background noise, salt and pepper noise represents sudden, isolated artifacts, and speckle noise captures spatial variations in tissue texture or structure (Khan et al., 2019). Additionally, for the functional scans, motion was induced by shifting the image volume over the time course, adding random degrees of motion to each subject’s image data. SNR and motion severity were computed both before and after adding noise and motion, and AIDAqc’s results regarding SNR in both the Chang and standard methods, as well as motion severity, were calculated.

#### 2.2.2 Outlier detection

To assess the reliability of the outlier detection, we applied the same as for quality metrics to introduce noise and motion, here only for one randomly selected scan from each sequence instead of all subjects, effectively creating single scans with simulated artifacts in the whole dataset (combined datasets 94_m_As and 94c_m_As). Thus, one scan at a time was induced with artifacts and then AIDAqc was applied 30 times on ~31 subjects (30 original, 1 artifact induced) each time with another scan as outlier.

### 2.3 Validation of outlier detection

#### 2.3.1 Initial validation approach

The validation of the outlier detection was conducted in two phases, addressing different aspects. In the initial phase, five experienced users manually rated the image quality of all datasets (Table 1). The individual images from the “manual_slice_inspection” folder (“static” images as part of the automatically generated output derived from the 3D/4D images), encompassing anatomical, diffusion, and functional MRI sequences, were examined. Raters identified potential outliers, that is, “bad quality data,” based on subjective evaluations of the image in comparison with all other images within one dataset. The level of agreement among raters when evaluating the same set of data for classifications, also known as the inter-rater agreement, was analyzed and compared with the results generated by AIDAqc. The classification features true/false positive (TP/FP) and true/false negative (TN, FN), sensitivity, specificity, and accuracy were calculated for quantitative comparison (Supplementary Table 3).

#### 2.3.2 Revised post hoc validation

Because of significant inter-rater variability in the initial approach, a revised and more comprehensive validation strategy was implemented. In this second phase, six experienced users conducted a detailed manual screening of the complete 3D/4D image files that AIDAqc identified as high-threshold outliers (AIDAqc voting thresholds of 4 and 5). Thresholds were introduced to create cutoff scenarios, in which the majority voting results are grouped into very strict (high threshold) or not as strict (low threshold) outliers. In a systematic way, different thresholds for the number of manual raters and AIDAqc outlier algorithms were compared to find an optimum (Supplementary Material: “Role of the rating threshold for the agreement between manual raters and AIDAqc”).

### 2.4 Statistics and visualization

The statistical analyses were conducted using GraphPad Prism version 9.5.1 (GraphPad Software, Boston, MA, United States,www.graphpad.com) and custom code written in Python version 3.11.4, with the libraries statsmodels (Seabold & Perktold, 2010) and scipy.stats (Virtanen et al., 2020).

Fleiss’ kappa score, a statistical measure utilized to assess the inter-rater agreement between multiple raters when categorizing items into different groups, was used to evaluate the reliability of manual ratings of image quality as “good” or “bad.” The interpretation of Fleiss’ kappa scores indicates various levels of agreement: scores less than 0 indicate poor agreement, 0.01–0.20 suggest slight agreement, 0.21–0.40 imply fair agreement, 0.41–0.60 signify moderate agreement, 0.61–0.80 denote substantial agreement, and scores between 0.81 and 1.00 represent almost perfect agreement.

Additionally, various data analysis and visualization tasks were done using the Python libraries pandas, numpy, matplotlib, seaborn, and nibabel. DTI and rs-fMRI postprocessing for selected datasets were conducted using an atlas-based approach with our in-house software AIDAmri (https://github.com/Aswendt-Lab/AIDAmri). Briefly, AIDAmri performs brain extraction and data correction (slice time correction, motion correction, spatial smoothing with full width at half maximum, and high-pass filtering for rs-fMRI), followed by a multistep registration with the Allen Mouse Brain Reference atlas (CCF v3). Functional connectivity is derived by a seed-based Pearson correlation among all atlas regions. Structural connectivity matrices are produced with deterministic fiber tracking using DSI Studio; for details seePallast et al. (2019).

### 2.5 Ethics statement

The data corresponding to 94c_m_As and 94_m_As fromTable 1were acquired in strict adherence to the ARRIVE guidelines for reporting in vivo animal experiments and the IMPROVE guidelines for stroke animal models, as recommended byKilkenny et al. (2010)andPercie du Sert et al. (2020). These datasets were processed following comprehensive ethical protocols approved by the Landesamt für Natur, Umwelt and Verbraucherschutz North Rhine-Westphalia, Germany, under animal protocol numbers 84−02.04.2016. A461 and 84−02.04.2014.A305. Further ethical clearance was obtained from the Gothenburg Ethics Committee, Sweden, under animal permit number 1551/2018. In the case of data sourced from other groups within the research, all animal experiments were approved by the respective local ethical authorities.

## 3 Results

### 3.1 AIDAqc workflow

AIDAqc was designed as a three-stage open-source pipeline of Python scripts (https://github.com/Aswendt-Lab/AIDAqc), which requires minimal user input (Fig. 1). The installation and application of AIDAqc were independently tested by four scientists and optimized using the datasets (see next section). T1, T2, DTI, and fMRI scans can be used—independent of the underlying MR sequence (spin echo, gradient echo, FLASH, etc.)—as input. AIDAqc accepts a vendor-specific data format by Bruker Biospin and the widely used vendor-independent BIDS/NIfTI data format. No prior data sorting is necessary as the main input folder will be iteratively searched. Each of the three stages can be run individually but the result of the third stage depends on the sequential processing of stages 1 and 2, respectively. The results are reported automatically in machine-readable csv files as well as figures and image sequences.

### 3.2 Dataset statistics

To test AIDAqc on a wide range of MRI setups, sequences, and animal models, 65 international MRI experts were invited as part of an initiative from the STANDARD working group of the European Society for Molecular Imaging (documented on GitHub:https://github.com/Aswendt-Lab/MRI_Standardization_AIDAqc).

**Fig. 5.**
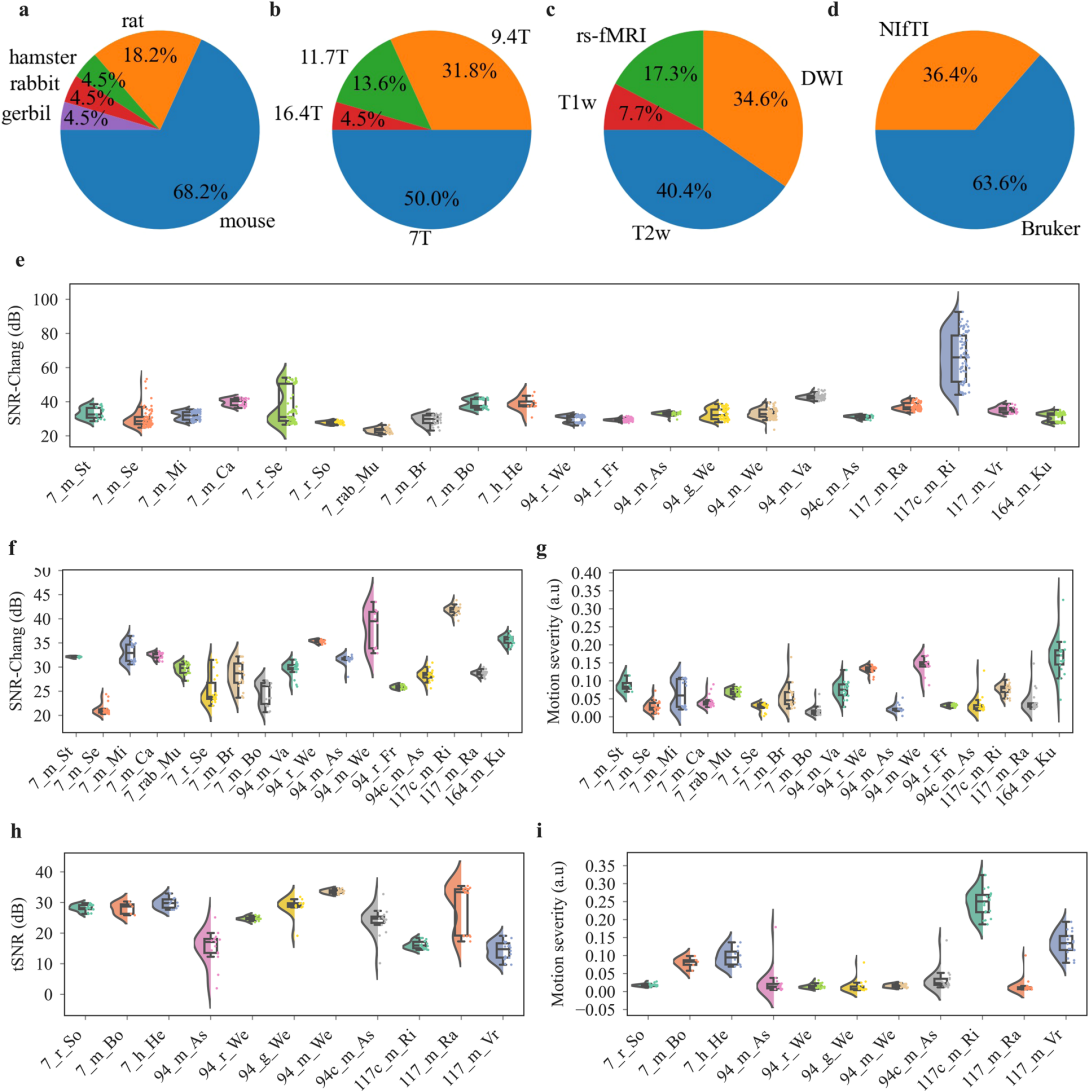
Summary of dataset distribution regarding basic statistical information (a–d) and quality features (e–i). Statistical distribution between all datasets regarding the (a) species, (b) field strength, (c) sequence, and (d) data format. In (d) “Bruker” refers to the Bruker raw data format and NIfTI to the Neuroimaging Informatics Technology Initiative data format. Summary of quality features for all (e) anatomical scans, (f–g) diffusion scans, and (h–i) functional scans. Note that the distribution of datasets for (e–i) is different, as not all datasets contain diffusion and functional scans in addition to anatomical scans.

Finally, n = 19 laboratories provided a total of n = 23 small animal datasets to provide datasets consisting of about 2,600 anatomical, 1,200 diffusion, and 700 functional scans. These datasets had not been prescreened for outliers and were used to test and validate AIDAqc. In applying AIDAqc to test its efficacy, each of the 23 datasets was processed independently. This separate analysis allowed us to maintain the integrity of the outlier detection process, ensuring that any identified quality issues were specific and relevant to each dataset’s unique characteristics. All data are publicly available (see section 5: “Data and Code Availability”). The majority of datasets were obtained with mice (68.2%) and rats (18.2%), and single studies used hamsters, rabbits, and gerbils (Fig. 5a). MRI was acquired with a broad range of hardware in terms of field strength (7–16.4T) and receive/transmit coil arrangement (Fig. 5b). Most studies were conducted at 7T (50%) followed by 9.4T (31.8%) using anatomical (T1- or T2-weighted MRI) and diffusion-weighted imaging (DWI), as well as resting-state functional MRI (rs-fMRI) (Fig. 5c). Most data were provided in the Bruker raw data format (63.6%) next to the NIfTI format of which only six datasets were BIDS-compliant (Fig. 5d).

The datasets further varied substantially in terms of FOV, matrix size, and image resolution (seeSupplementary Fig. 1and extended version ofTable 1inSupplementary Table 1). In addition, we also applied AIDAqc in a proof-of-concept approach to two abdominal datasets (Supplementary Fig. 2) and previously published large repositories containing mouse and rat data (Supplementary Table 2). The AIDAqc output is summarized in the online repository (https://doi.gin.g-node.org/10.12751/g-node.q82cjj/), featuring detailed reports for SNR, tSNR, and motion severity.

The mean SNR in anatomical scans (neuroimaging datasets as listed inTable 1), as calculated using the Chang method, was 32.1 dB ± 4.6 dB (Fig. 5e). For the diffusion scans, the mean SNR was 28.4 dB ± 5.0 dB (Fig. 5f), and in functional scans, we measured 27.4 dB ± 5.9 dB tSNR (Fig. 5h). Temporal SNR across functional MR sequences varied with large differences in variance between specific studies, for example,*117_m_Ra*, which was acquired with two different sequences, visible as two tSNR populations (Fig. 5f). The motion severity was rather stable for 16 out of 18 diffusion and 9 out of 11 functional studies, respectively (Fig. 5g, i). The motion severity varied between 0.017 (7_m_Bo) and 0.217 (7_m_Lo) in diffusion scans and between 0.002 (117_m_Ra) and 0.323 (117c_m_Ri) in functional scans, respectively.

Initially, the intention was to also apply AIDAqc to a published large, multicenter dataset to demonstrate the tool’s adoption and efficacy (seeSection 4.3). However, it became apparent that these datasets had already undergone screening for poor-quality data, rendering them unsuitable for our intended purpose. As a result, we also applied the tool to all NIfTI datasets fromTable 1in a*collective manner*(Supplementary Fig. 8), and outliers in addition to the normal statistical outliers were registered in the*voting.csv*through the majority vote approach.

### 3.3 Automated SNR measurements

#### 3.3.1 Comparison of SNR across datasets

**Fig. 6.**
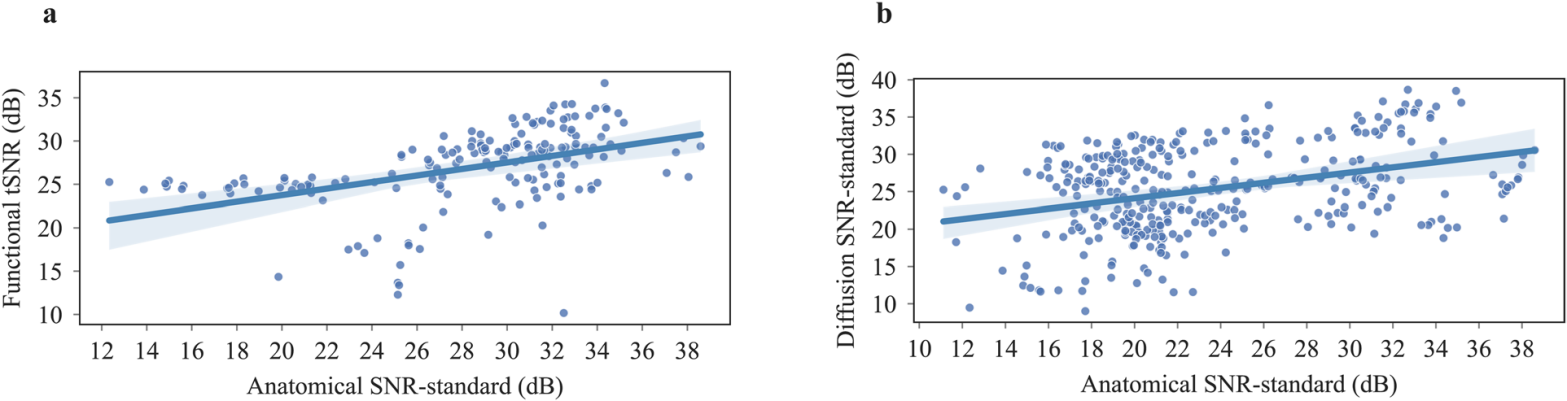
Scatterplots (a) Spearman correlation between functional tSNR and anatomical standard SNR. (b) Spearman correlation between diffusion standard SNR and anatomical standard SNR of subjects who had both scans.

Given the differences in MRI hardware (scanner, coils), sequences, shimming procedure, and animal experiment, here it was not meaningful to statistically compare all individual datasets. Importantly, the effects of differences in MRI hardware (gradients, coils) overrode the positive correlation of field strength and SNR (Fig. 5). In studies selected for a relatively homogeneous voxel volume of 9.5 nl ± 1.2 nl, there were significant differences in SNR values for different magnetic field strengths, also for selected studies with the same magnetic field strength and an increasing voxel volume, there were significant differences observed. As expected, SNR was scaled with magnetic field strength and voxel volume (Supplementary Fig. 3). Within studies including both anatomical and functional scans, SNR and tSNR correlated positively (Spearman r = 0.57, p < 0.001) (Fig. 6a). A similar positive correlation was found between anatomical SNR and diffusion SNR (r = 0.30, p < 0.001) (Fig. 6b).

**Fig. 7.**
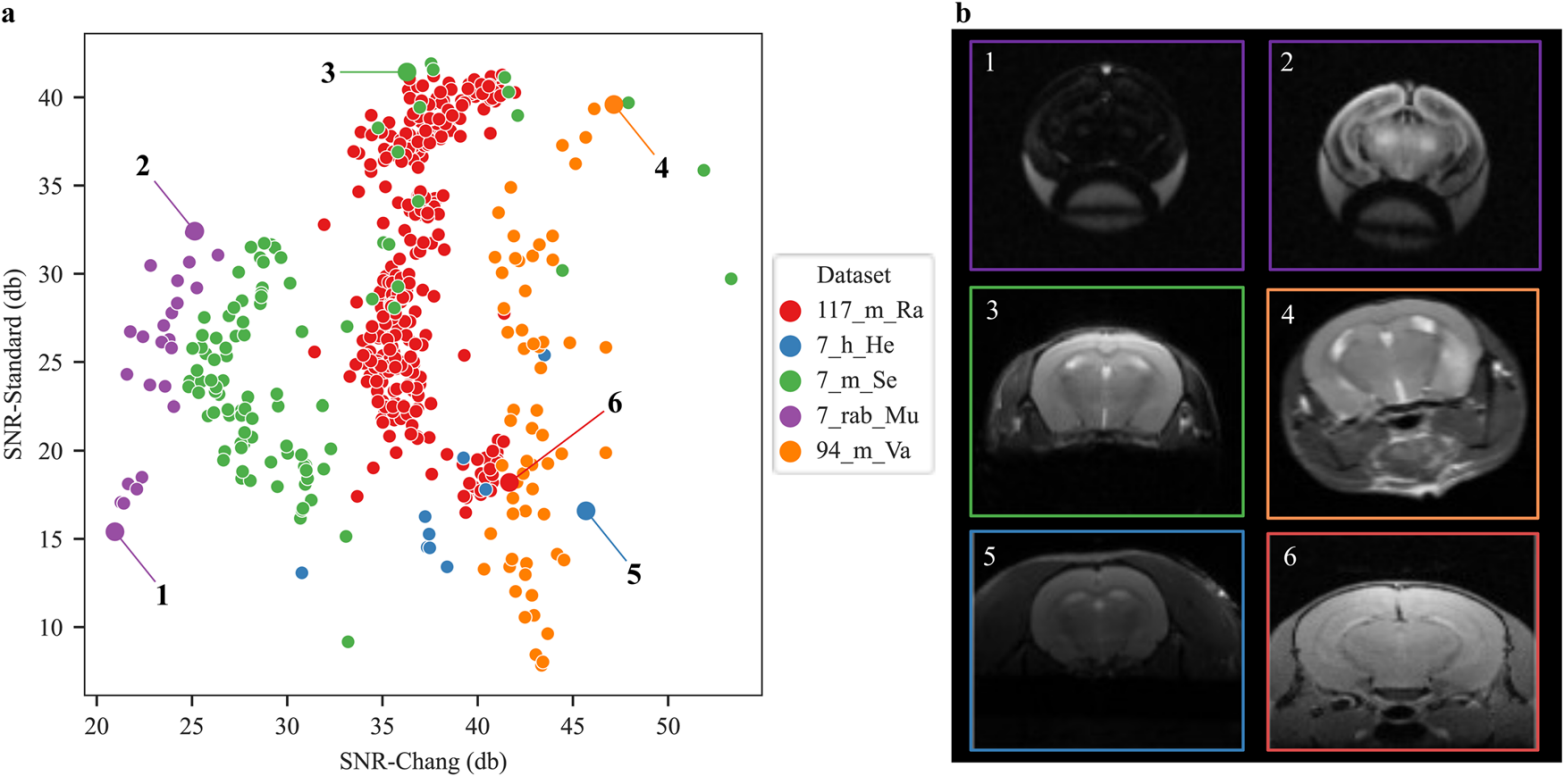
Scatterplot of the relationship between SNR values calculated using the Standard and Chang method. (a) SNR-Standard and SNR-Chang comparison for five randomly selected datasets (7_h_He, 7_m_Se, 7_rab_Mu, 94_m_Va, and 117_m_Ra). (b) Selected images from the different datasets reflect similar SNR (examples 1 and 4) and different SNR (examples 2, 3, 5, and 6).

We further quantitatively compared the two SNR methods (SNR-Standard vs. SNR-Chang) for all anatomical datasets (Supplementary Fig. 4). The SNR methods correlated positively (Spearman r = 0.16, p< 0.001). For a randomly chosen selection of five datasets, we further validated that the quantitative SNR values represent the qualitative “visual” appearance of the images, that is, higher SNR corresponds to more brightness and contrast in the image (Fig. 7a, b, image #3–4). The visual comparison (Fig. 7b) also suggests that SNR-Chang is less sensitive for images with brightness differences (Fig. 7b, #1–2). The SNR-Standard method, however, can be falsified when the ratio of subject to field of view is high (Fig. 7b, #5–6).

### 3.4 Artifact simulation to evaluate the reliability of quality metrics and outlier detection

#### 3.4.1 Quality metrics

**Fig. 8.**
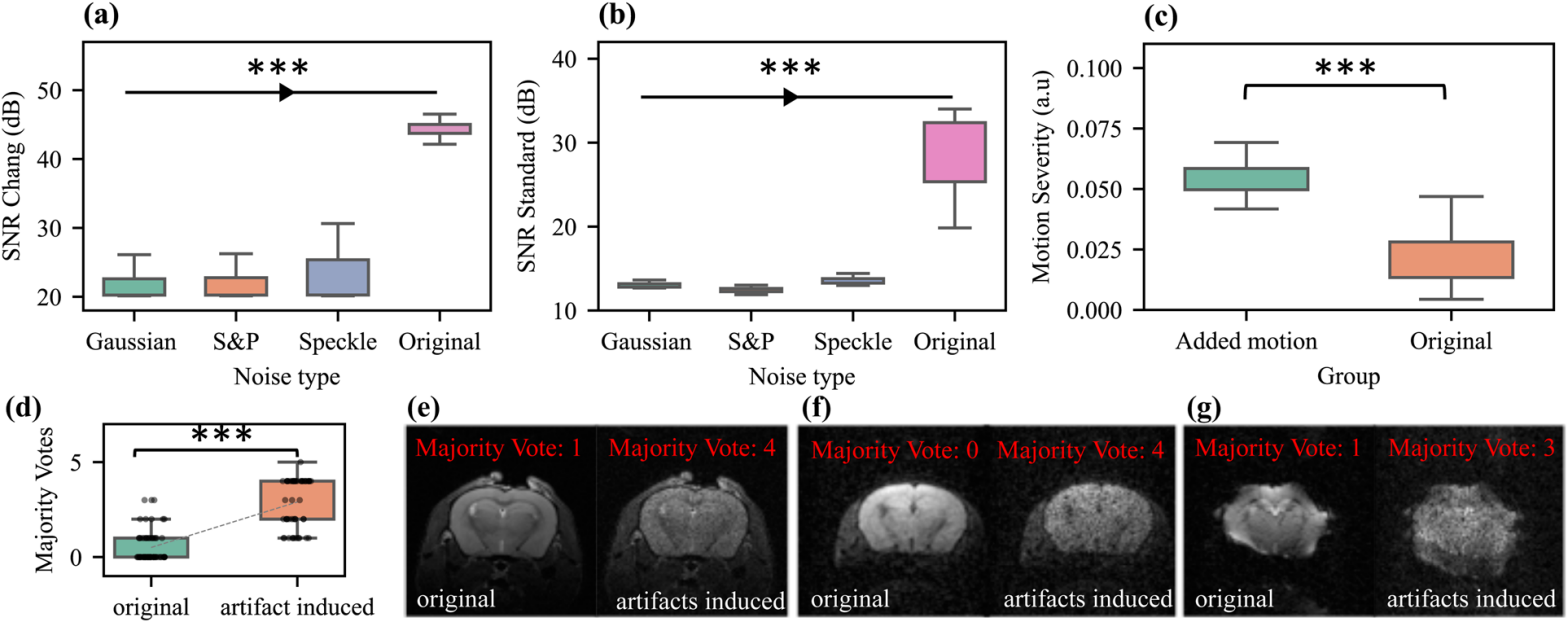
Artifact simulation to evaluate the reliability of quality metrics and outlier detection. Comparison of signal-to-noise ratio (SNR) and motion severity metrics between the induction of different noise types and motion versus the original image in (a) SNR-Chang, (b) SNR Standard Method, and (c) Motion Severity. The boxplots illustrate the distribution before (original) and after noise addition and motion induction. Significant differences (*** indicates p < 0.001) were observed between each noise-induced group and the original group for using Welch’s*t*-test. The evaluation of majority votes on artifact-induced images revealed significantly higher votes compared to the original images (*** indicates p < 0.001). (d) Consistent higher majority votes in the artifact-induced images compared to the original. Representative anatomical (e), diffusion (f), and functional (g) images before and after addition of artifacts with related majority votes.

The analysis of data with simulated (added) Gaussian, salt and pepper, and speckle noise (Fig. 8a, b) revealed a significantly lower SNR compared with the original image using both SNR methods. Similarly, adding motion to the rs-fMRI scans significantly increased the motion severity index (Fig. 8c). Welch’s*t*-test was applied for comparison, indicating significant differences (p < 0.001) between the modified and the original group data for each metric.

#### 3.4.2 Outlier detection

In the approach to showcase the reliability of outlier detection and majority voting, a significant difference in majority votes between the artifact-induced and original images was detected (p < 0.001), with the artifact-induced images consistently receiving higher majority votes.

Representative examples show that the majority vote was consistently higher in the artifact-induced images than in the original (Fig. 8d). For the anatomical image, the number of ML algorithms flagging the original image as an outlier was 1, whereas in the contaminated counterpart image, it increased to 4 (Fig. 8e). Similarly, in the case of the diffusion sequence, the majority vote was 0 for the original image and increased to 4 for the contaminated image (Fig. 8f). In functional sequences, the contaminated image was flagged with a majority vote of 3 compared with 1 for its original counterpart (Fig. 8g).

### 3.5 Validation of outlier detection

#### 3.5.1 Initial validation approach

**Fig. 9.**
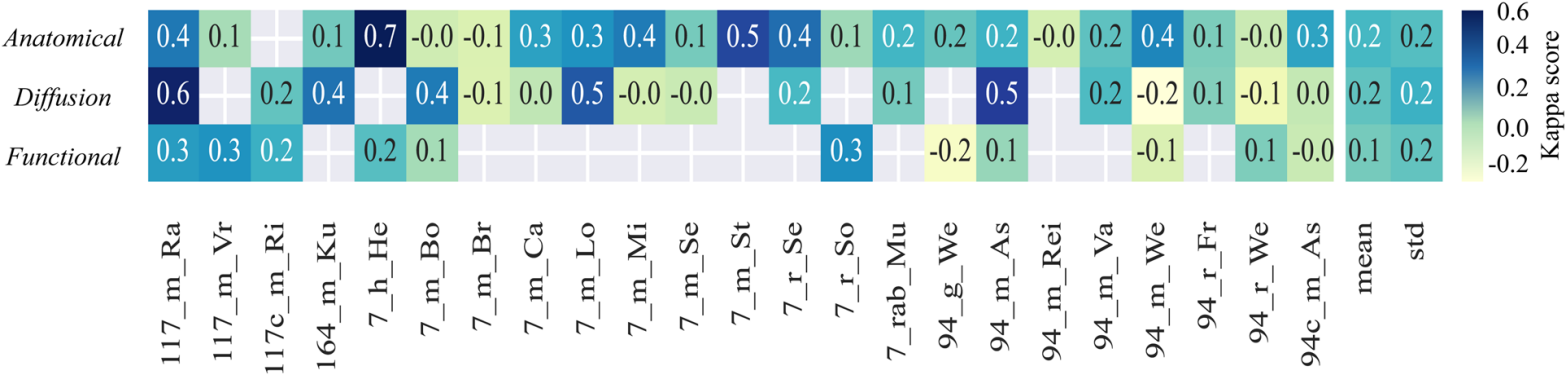
Inter-rater agreement. Fleiss kappa scores were calculated separately for each dataset, serving as an indicator of inter-rater agreement. The last two columns present the mean and standard deviation (std).

To gain a ground truth for the comparison of classification, we worked with the votings for all datasets from n = 5 experts with 5–10 years of experience in small animal MRI. The experts were asked to identify bad-quality images in the 23 datasets including 4,452 images. The assessment was purely objective and referred to visible artifacts or noise in the images, without any further specific step-by-step evaluation. As the main result, the mean inter-rater agreement for all data (Fig. 9) was low across anatomical, functional, and diffusion scans (mean Fleiss Kappa score 0.2 ± 0.2, 0.2 ± 0.2, and 0.1 ± 0.2). For the datasets with a substantial inter-rater agreement, for example, 7_h_He and 117_m_Ra (Fleiss Kappa score >= 0.6), and with no inter-rater agreement, for example, 7_m_Br and 94_m_Rei (Fleiss Kappa score 0.0), respectively, there was visually no related identifiable image feature or pattern.

In addition, we conducted a comprehensive analysis using a confusion matrix (Supplementary Material Table 3and related text). This analysis was based on calculations of sensitivity, specificity, and accuracy, considering various threshold scenarios between the AIDAqc and human raters (Supplementary Fig. 5). Here, a threshold relates to a minimal number of positive votes. The threshold = 3 for AIDAqc voting refers to the majority vote of 3, that is, at least three algorithms labeled the data to be of bad quality. Similarly, the threshold = 3 for the manual raters indicates that a file was considered of bad quality by at least three of the expert raters. The highest sensitivity (0.20 ± 0.28), that is, out of all the actual “bad-quality” datasets, how many were identified by AIDAqc as “bad-quality” data, was reached with the AIDAqc threshold set to 1 and the manual rater threshold set to 4. For this threshold combination, the overall classification accuracy (0.79 ± 0.13) and specificity (0.81 ± 0.12), that is, identification as “no bad quality,” were high. Despite these efforts, the low inter-rater agreement rendered the results less definitive.

#### 3.5.2 Revised post hoc validation

In response to the low inter-rater agreement observed in the initial validation phase, a second, more focused validation approach was implemented. In this revised strategy, the same experts were tasked with validating only those images flagged as high-threshold outliers (voting thresholds of 4 and 5) by AIDAqc. The precision measurements, which reflect the proportion of images correctly identified as outliers by AIDAqc and confirmed by the experts, showed an average precision of 70.72 ± 9.9%, with an adjusted precision of 72.23% when unsure cases were excluded.

### 3.6 Relevance of outlier removal for data postprocessing

**Fig. 10.**
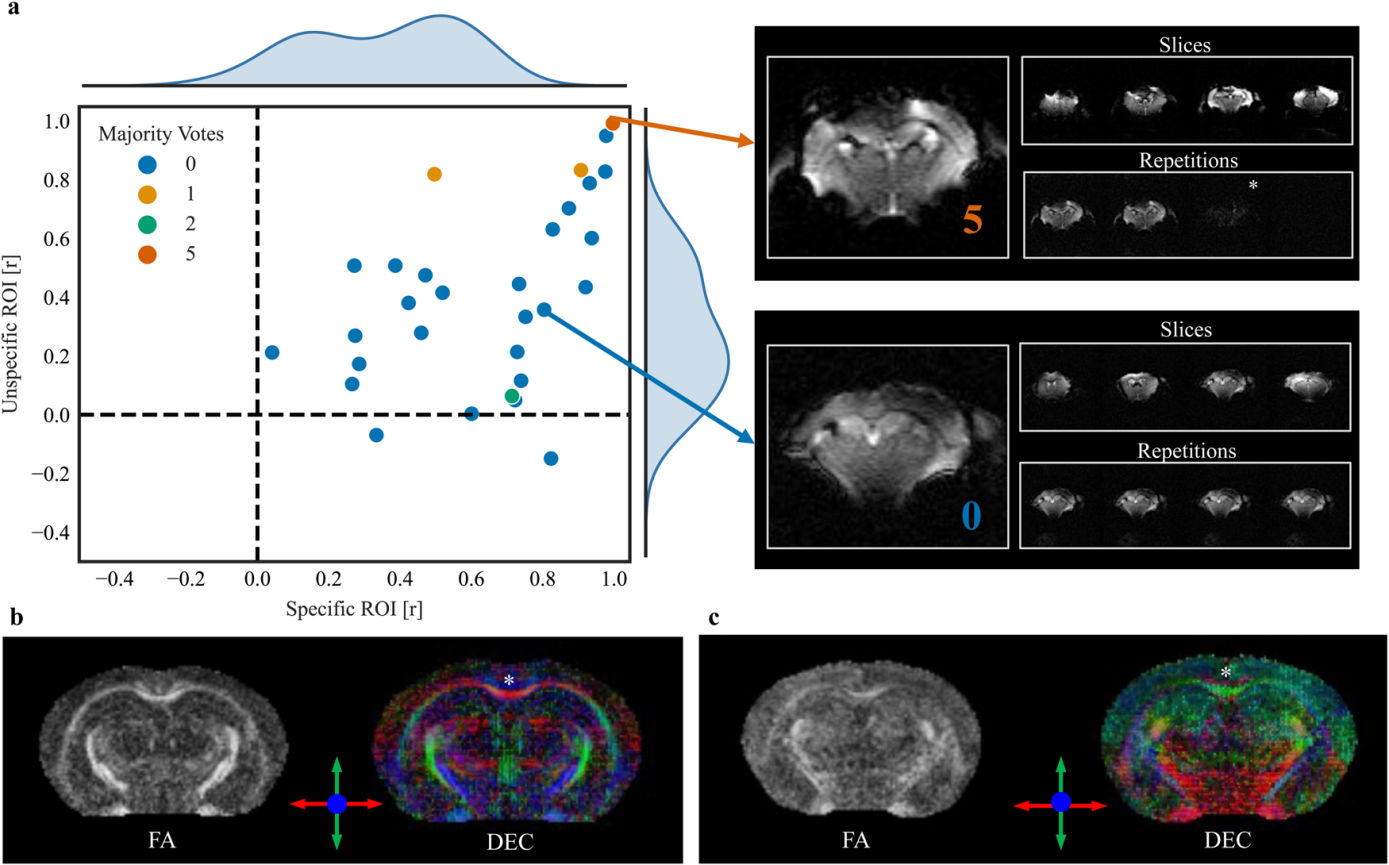
Functional specificity and diffusion tensor imaging analysis for quality assessment. (a) Functional specificity analysis comparing the functional connectivity with the primary somatosensory barrel field relative to the contralateral homotopic area and the anterior cingulate area (ACA). The X-axis represents the correlation to the specific ROI (contralateral primary somatosensory barrel field), and the Y-axis represents the correlation to the unspecific ROI (ACA). Expected results include high correlations for the specific ROI and low correlations for the nonspecific ROI. Data points across the dataset reveal correlation values for each ROI, with outliers, identified by AIDAqc with a majority vote, showing unusually high correlations for the nonspecific ROI. This suggests that these outliers likely represent poor-quality data, which can adversely affect functional connectivity measures. (b, c) Two different examples of diffusion images are classified as good versus bad quality using AIDAqc. Whereas similar anatomical structures can be identified in the FA map, the diffusion tensor (displayed as direction encoded color map) shows very different and anatomically incorrect tensor orientations, for example, in the corpus callosum (*) connecting both hemispheres with horizontal (red) fibers crossing the brain midline.

To assess the importance of identifying poor-quality data through AIDAqc in subsequent postprocessing steps, we processed functional and diffusion datasets from two representative control groups, 94c_m_As and 94_m_As, using our in-house software AIDAmri, as established in a prior study (Pallast et al., 2019). These datasets were chosen because they represent control animals, that is, no disease or intervention model, to provide a consistent baseline for assessing functional and structural specificity. In terms of SNR, the functional specificity calculated for the merged dataset (Fig. 10a) illustrates how connectivity metrics can differ significantly even when images appear visually similar at first glance. We assessed functional specificity by computing correlations between specific and nonspecific ROIs. The X-axis of the scatter plot inFigure 10adetails the specific ROIs, which include the left and right primary somatosensory barrel fields, expected to show high correlations. The Y-axis represents the nonspecific ROI, formed by combining the anterior cingulate area with the left primary somatosensory barrel field, expected to show lower correlations (Grandjean et al., 2023). High values for the nonspecific ROI might suggest problems either in the unprocessed data or during the processing steps. Adjacent to the scatter plot, the subject with a majority vote of 5 and one other subject not flagged by AIDAqc are shown representatively. In case of a majority vote of 5, a complete loss of signal in the last 25 repetitions was detected, potentially due to a gradient malfunction, leading to incorrect bias field correction and poor atlas registration (Supplementary Material Fig. 7). Among the subjects from the merged datasets, this subject’s data confirm that image quality issues predominantly originate from the data itself, not the processing steps, as indicated by AIDAqc’s findings. This emphasizes that while high correlations for nonspecific ROIs could stem from various sources, in this instance, the problems are specifically related to data quality. In diffusion tensor imaging data (Fig. 10b, c), artifacts may not be detectable by examining single slices or directions alone. Instead, diffusion analysis can produce markedly different results even when visual image quality appears only slightly varied. For instance, in the data identified as an outlier (Fig. 10c), marked by a majority vote of 3 out of 5 algorithms, the diffusion tensor distribution showed a more random pattern and less anatomical coherence compared with the good-quality dataset. Notably, key structures like the corpus callosum, a major fiber tract connecting both hemispheres, were detected in the good-quality image but not in the outlier data. These observations are supported by a comprehensive qualitative comparison across all subjects from the two datasets, showing a higher majority vote of 2 and 3 related to suboptimal FA (fractional anisotropy) maps. Extended qualitative comparisons of diffusion-weighted images and corresponding quality metrics are detailed inSupplementary Figure 6.

## 4 Discussion

AIDAqc was developed with the aim of automatically identifying bad-quality data from large and heterogeneous small animal MRI datasets. Although classification was implemented as an automated selection using statistics and machine learning, it should not act as a black box. On the contrary, it was designed to report the classification transparently and flag poor-quality data that need further inspection by the user, for example, it can still be useful for a particular purpose.

To achieve these goals, we collaborated with n = 19 international MRI laboratories, which shared mouse, rat, and gerbil datasets. This collaboration ensured a diverse testing environment, encompassing a wide range of scanner hardware, imaging protocols, and experimental conditions (a detailed summary of the datasets can be found inSupplementary Material Fig. 1andTable 1). In an iterative process, the AIDAqc workflow was adjusted and optimized to facilitate the processing of all acquired datasets. The findings of this study highlight the potential for improvement in quality control, as well as the benefits of automating the process, thereby avoiding manual interventions and reducing related errors. The AIDAqc pipeline was developed in Python as an open-source project, to allow full flexibility to include other metrics or automated calculations and integrate AIDAqc in other pipelines by the small animal imaging community.

### 4.1 The importance of QC/QA in small animal MRI

Conceptually, QC and QA describe ongoing efforts to ensure the stability of the MRI scanner and the assurance of homogeneous image quality (Sreedher et al., 2021). Small animal MRI has not adopted clinical and industry standards so far, to a certain extent because of the belief that such standards would limit scientific freedom and innovation (Wijnen et al., 2023). In contrast, we argue here in line with the community-driven standardization initiatives as part of the imaging societies ESMI, ESMRMB, and ISMRM, which standards are required to enhance the reliability and reproducibility of all neuroimaging studies and are necessary in the translation of preclinical to clinical protocols.

A central element of clinical QC is checkpoints defined before the project starts, to avoid poorer quality data passing on to the next processing stage (Strother, 2006). Such strict exclusion criteria limit retrospective cherry-picking and avoid bad-quality data to introduce false positive and/or negative results (Niso et al., 2022). However, most animal studies still favor an explorative approach, for which a retrospective selection of good-quality data as implemented in AIDAqc is more meaningful. Similarly, for reuse of data and, more importantly, for reproducibility of existing studies that rely on large databases (e.g., OpenNeuro), retrospective identification of image quality in general and, in particular, signal-to-noise ratio (SNR) and image artifacts is critical. AIDAqc fills an existing gap of efficient software tools and overcomes manual bad-quality selection, which in most cases is bound to an individual decision by the researcher. Importantly, AIDAqc does not contradict previous standardization efforts but complements the development of reliable preclinical MR assays that lead to comparable results across laboratories (Doblas et al., 2015;Waterton et al., 2019).

### 4.2 Outlier detection based on quality metrics

#### 4.2.1 Rationale of selected quality metrics

The selection of bad-quality data for different MR sequences cannot be accurately done by a single quality measure. Similar to QC tools for human MRI (Esteban et al., 2017;Raamana, 2018;Williams et al., 2023), AIDAqc relies on the combination of multiple automatically derived image quality measures, that is, (t)SNR, motion, and ghosting. The combination ensures high versatility for multiple MR sequences and mitigates known disadvantages for a single quantitative assessment of image quality, as described for SNR, for example (Erdogmus et al., 2004). We have selected basic quality metrics well established in the MRI community. As a novel aspect, we introduce automated calculations, which do not require manually drawn regions of interest. With simulated image artifacts, we have shown that these calculations provide a good differentiation from image data without artifacts.

It should be noted that this approach could be extended in future versions to provide more information about the type of artifact (e.g., the discrimination of discrete vs. Nyquist ghosts) and include other metrics described in the literature, for example, the contrast-to-noise ratio (CNR), root-mean-squared error (RMSE), coefficient of joint variation of gray matter and white matter, full-width half-maximum estimation of the blurriness of the image, and the overlap of tissue probability maps estimated from the image and a template (Chow & Paramesran, 2016;Esteban et al., 2017). These quality metrics were not included as the information content either overlaps with integrated metrics (e.g., SNR and CNR) or segmentation and template registration, respectively, are required. We decided against a segmentation or template registration process as this would increase processing time and unnecessarily limit the input images to a specific format and orientation. Other restrictions should be considered as well, such as in the case of RMSE where the reliance on a specific reference image or ground-truth data would pose practical challenges due to the diverse nature of MRI datasets and the absence of universally applicable reference images. In the case of CNR, where the signals from two regions of interest are subtracted and divided by noise, automated analysis is theoretically feasible. However, generalizing this approach across different datasets is challenging due to variations in imaging sequences and scan orientations.

#### 4.2.2 Synergistic application of two SNR methods

In this study, we compared two distinct methods for assessing SNR without the need to manually label regions of interest: the noise estimation method developed byChang et al. (2005)and the standard SNR method extended with an automatically created center of intensity to measure the signal and cuboids in the corners to measure noise. The results of the quantitative comparison across all datasets revealed the strengths and limitations of each approach, which can be complementary and used as an advantage to enhance the reliability of SNR measurements through a synergistic combination as it is done in AIDAqc. The standard method excels in capturing spatial variations in image quality, particularly in regions of interest where signal integrity is paramount, while Chang’s SNR method provides a statistical framework for noise estimation. For datasets with a high probability that the subject is not in the center of the field of view, results showed that the Chang approach is more trustworthy. In the comparison of five randomly selected datasets (7_h_He, 7_m_Se, 7_rab_Mu, 94_m_Va, and 117_m_Ra), the distribution along the standard method is higher than the SNR-Chang method across each dataset. This variation in standard deviation across different datasets suggests that SNR-Chang would better distinguish data from different sources in a scenario combining different studies. Also through the simulation tests of noise and specifically applying the gamma function to change intensity and contrast, mimicking shadows in MRI images, we identified the Chang method to be more responsive to general intensity changes compared with merely adding different types of noise. However, by focusing on one modality or similar images of a dataset, the SNR standard can create a better distinction. Whether this distinction is desired or not depends on the research question at hand. Therefore, it is beneficial to consider the SNR methods first from a synergistic viewpoint. Overall, we found that this synergistic approach enhances the accuracy and robustness of SNR measurements, and subsequently, the machine-learning algorithms can use both features as their input, providing researchers with a more comprehensive understanding of image quality, particularly in neuroimaging studies where image quality directly impacts the validity and interpretability of results. The SNR assessment was also validated qualitatively and quantitatively by simulating noise.

#### 4.2.3 Detection of motion and ghosting using mutual information

Motion was quantified with a novel application of mutual information (MI). This approach was validated by introducing random motion into the dataset. Incorporating MI for assessing motion severity and ghosting was inspired by the recognized effectiveness of MI as an image registration metric (Maes et al., 2003). The strength lies in the ability of MI to capture nonlinear relationships and tolerate intensity variations. These intensity variations are particularly prevalent in preclinical animal models, where images often feature strokes and lesions. Further investigation into the effectiveness of mutual information in addressing motion severity and ghosting, considering various artifact characteristics and preprocessing techniques, would be valuable for optimizing artifact assessment. In future studies, this method could be compared and complemented by other machine-learning approaches to detect motion (Fantini et al., 2021;Lorch et al., 2017), which have so far only been used in human MRI. The discrimination of the types of ghosts is also planned in future versions of AIDAqc.

### 4.3 Majority voting

As a novel concept, we introduce a statistical- and machine-learning-based outlier detection method in the form of majority voting. In the purely statistical way to determine outliers based on the interquartile range (Vinutha et al., 2018), a single feature (single variate) of image quality is used. Incorporating a multivariate outlier detection algorithm can lead to more accurate and general results. Using multiple algorithms together and creating a majority vote for an outlier can even stabilize the results further as each algorithm is selective for different types of quality measures and data. With simulated image artifacts, we have shown the robustness and reliability of the majority voting approach in identifying outliers.

The chosen machine-learning algorithms ocSVM, IF, LOF, and EE have been successfully used, for example, in MRI-based tumor classification, and tissue matter segmentation (Mayer et al., 2022;Mohammed et al., 2021;Ong et al., 2022;Zhang, 2011). However, the concept of bringing all of these outlier detectors together and using them as part of a majority vote in QC has, to our knowledge, not been used before. The adaptable and open-source structure of AIDAqc enables the seamless integration or removal of ML algorithms to adapt to specific requirements and new research results. Exploring the integration of additional algorithms such as autoencoders and robust principal component analysis into the majority approach could offer avenues for enhancing its efficacy. These algorithms bring unique capabilities such as nonlinear feature representation and an enhanced resilience to noisy or incomplete data, potentially enriching the diversity of perspectives considered in the decision-making process. However, it is essential to acknowledge that such augmentation might incur heightened computational demands and processing time. Nonetheless, investigating the interplay of diverse machine-learning techniques within the majority voting framework holds promise for advancing its performance.

We noticed that the thresholds to set the minimal number of manual raters and outlier algorithms rating data to be “bad quality,” respectively, have a huge impact on the validation of the classification. To understand the role of AIDAqc in outlier detection, it is essential to recognize its capability to identify subjects who deviate from the provided cohort. This does not necessarily imply that flagged subjects are inherently of poor quality or that the specific artifacts cannot be reduced during postprocessing, for example, using motion correction. In a scenario where a cohort consists of subjects selected from two or more different datasets with different acquisition parameters, AIDAqc would not classify an entire group as an outlier, but rather identify individual subjects who have characteristics that differ from their respective group. This targeted outlier detection is a significant advantage of AIDAqc, rooted in its machine-learning algorithms tailored for outlier identification. Traditional statistical approaches often falter in such complex scenarios. Therefore, when designing studies or research questions involving data from multiple sources, leveraging AIDAqc across all datasets*collectively*becomes justified, serving to refine the application of this tool according to the specific research objectives.

In practice, determining the majority vote threshold should involve subsequent checking of the data. As illustrated in the example of a functional scan with a high majority vote indicating a potential issue (Fig. 10a), detailed inspection revealed that the high voting was related to disruptions in the last 25 repetitions due to a potential gradient malfunction. Removing these affected repetitions could rectify the issue, illustrating how the majority voting approach assists in pinpointing specific problems for targeted corrections.

When using AIDAqc to detect outliers, a balance between sensitivity and specificity is critical. Users retain the flexibility to adjust the threshold for the majority voting based on the desired balance between these two metrics. For example, opting for a threshold of a majority vote of 3 or higher may lead to an increase of false positives, thereby reducing specificity, but could potentially decrease false negatives, consequently increasing sensitivity. Conversely, selecting a more stringent threshold, like only those subjects with a majority vote of 5, might further diminish false negatives, increasing the specificity but lowering the sensitivity. After the initial process, researchers can simply focus on the data with the highest majority votes or adjust the threshold based on their preferences.

### 4.4 Validation of outlier detection

#### 4.4.1 Initial validation approach

**Fig. 11.**
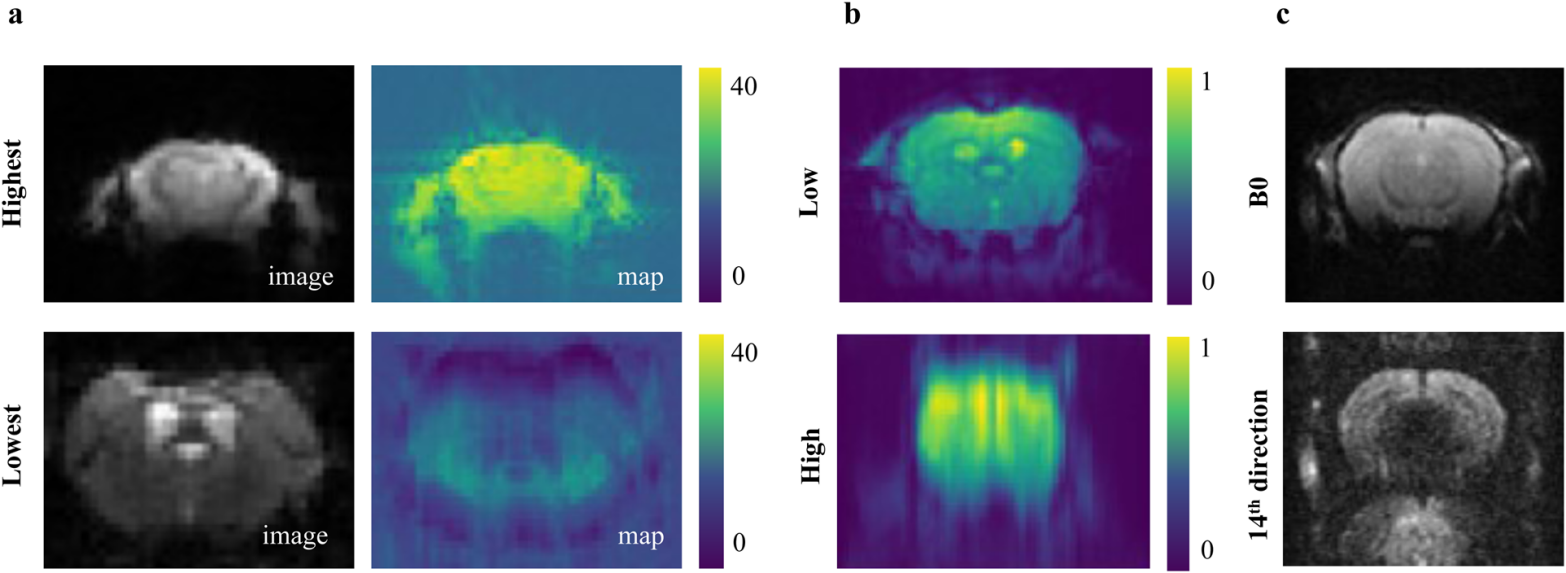
(a) rs-fMR images and their corresponding tSNR maps selected by the highest and lowest values calculated by AIDAqc. (b) Low and high motion examples for rs-fMR image volumes averaged over time to visualize the motion. (c) Ghosting artifacts in the 14th diffusion direction of the 4D diffusion image volume of one subject. Ghosting artifacts can be seen in the lower image, where the upper image corresponding to another the first diffusion direction of the same image volume was rated by the users, and as it does not show any particular problem, it was not rated as a bad-quality image. (a, b) were selected based on the highest (best) and lowest (worst) values between all datasets fromTable 1.

Visual criteria of image quality are not standardized and are strictly user dependent. It was, therefore, not surprising to see a significant difference in user ratings for the individual datasets. However, we questioned the rather poor confirmation of AIDAqc outlier detection by the expert raters. Finding a common ground truth proved to be a very challenging task. We noticed that very important noise and image artifact components in the datasets remained hidden for manual raters and can only be identified by automated algorithms scanning the full 2D/3D/4D image volume. Visually detectable artifacts might exist in other slices or in the case of functional and diffusion scans, artifacts might exist at a different time point and diffusion direction, respectively. A tSNR map, which was not part of the manual evaluation, might have been sufficient for manual raters to see the difference between two rs-fMR images (Fig. 11a). Similarly, it is very unlikely to detect motion and ghosting from inspection of single images, as the motion between images requires setting a temporary reference image (Fig. 11b), and ghosting might appear only in specific diffusion directions (Fig. 11c). That was also reflected in the sensitivity, which was by trend highest in anatomical compared with diffusion and functional datasets, that is, in the data which can be rated best manually. We noticed that the validation of the automated outlier detection based on the single images, produced as part of the AIDAqc output, remains the most feasible but not optimal scenario, especially for 4D scans. We, therefore, come to the same conclusion as others in human MRI that the manual evaluations are impractical or infeasible (Bedford et al., 2023;Chow & Paramesran, 2016), as it is not possible and not expedient to go through all the individual images for very large datasets.

Therefore, understanding the optimal combinations of thresholds for both AIDAqc and manual raters was crucial. For each sequence type (anatomical, diffusion, and functional scans), we identified the best combinations of thresholds for AIDAqc and manual raters. For the most important measure, sensitivity, that is, the rate of “bad-quality” data identified by AIDAqc compared with the manual raters, the optimal threshold for AIDAqc was 1. This might be interpreted as a logical choice for several reasons: (i) the error rate of AIDAqc is not fluctuating as it is the case for the objective classification by the manual raters, (ii) AIDAqc systematically evaluates every voxel of an image volume, each AIDAqc rater can be considered to have its unique strengths and weaknesses in identifying outliers. In contrast, manual raters, relying on visual inspection, do not have the same systematic approach. Thus it is worth checking any dataset, even if it only got one vote to be an outlier from AIDAqc. In contrast, manual rater thresholds exhibited a favorable trend for accuracy but not for specificity and sensitivity, likely influenced by poor inter-rater agreement.

#### 4.4.2 Revised post hoc validation

Consequently, we transitioned to a more focused validation method, as elaborated in the second stage of our process, providing a more targeted validation approach. indicating some variability in their assessments, yet demonstrating a generally consistent agreement with AIDAqc. This approach provided a more nuanced insight into AIDAqc’s outlier detection capabilities. By focusing on the high-confidence outlier predictions and considering the more decisive assessments of the experts, this method offered a better understanding of the effectiveness in identifying true cases of poor-quality datasets. The observed standard deviation underscores the inherent subjectivity in manual assessments, reinforcing the value of automated tools in systematically evaluating image quality.

### 4.5 Limitations

#### 4.5.1 Datasets

To validate the reliability of bad-quality detection using AIDAqc, we collected largely heterogeneous data in terms of animal, MRI hardware, and sequence. Unlike previous standardization efforts that focused on homogenization of data collection (Doblas et al., 2015;Grandjean et al., 2023;Waterton et al., 2019), wide variability was desired for probing the versatility of AIDAqc. Nevertheless, the heterogeneous datasets imposed a challenge for the analysis. This included automated sequence type recognition and the correct sorting into anatomical, diffusion, and functional scans, respectively, which were corrupted by nonstandard abbreviations for MR sequences (seeSupplementary Fig. 9a, b, d). As most sequences can be used for multiple purposes, for example, echo planar imaging (EPI) in both diffusion and functional imaging requires more in-depth metadata extraction to identify the actual type of scan. Such metadata extraction is in the current AIDAqc version only possible for BIDS or Bruker data but not for the NIfTI format as it contains only very limited metadata. In future versions, compatibility with DICOM data could be implemented because of its wide distribution in human MRI as well as other vendor-specific raw data next to the Bruker format to increase the versatility. AIDAqc uses an integrated list of strings of the most common nomenclature as keywords, for example,*turbo*,*rare*,*rest*,*diff*,*rs-*,*func*,*anat*,*struc*,*dwi*, and*dti*. Additionally, for DWI and functional images in the raw Bruker format, AIDAqc checks whether a gradient/b-value table is available to make the distinction. We cannot exclude, however, the possibility that automated parsing and sorting may fail for other unconventional sequence names. In the case of BIDS structure, for example, in the*7_rab_Mu*and*94_r_Fr*datasets, the NIfTI format is complemented with metadata in a standardized json file. To facilitate standardization, we highly recommend the BIDS format as it solves the issue of variable sequence names and dataset hierarchy of groups and time points as well as metadata information.

Another challenge was imposed by a corrupted image matrix logic in the x-y-z regime, for example, in the form of 256 x 40 x 256 imposing a x-z-y structure with z being the number of slices and x-y being the image dimension. This occurred in single datasets in both the raw data and by converting the data into the NIfTI format and required manual correction. Manual spatially reorganization is also necessary for noncoronal acquired datasets (seeSupplementary Fig. 9c, e, g). This step is critical, as the subsequent processing depends on the correct association of 2D/3D/4D sequences and scans. In future AIDAqc versions, automatic extraction of the actual image orientation and position of the animal would reduce this potential error and manual data preparation for the users. Another potential limitation is the fixed parameter setting in some of the outlier detection algorithms, which should be optimized if AIDAqc is to be used for completely different datasets.

#### 4.5.2 Automated feature calculations

Unlike most approaches to calculating SNR region based, which requires manual user input to draw the regions of interest, here two fully automated SNR calculations were implemented. This way, there is no need for the user to manually define regions, which makes the calculation less error prone and faster. We noticed, however, that the automated SNR calculation has some limitations. The standard SNR calculation is sensitive to the location of the subject in the field of view: (i) when the subject fills most of the field of view, especially the corners, the cuboids for calculating the standard deviation cover not only noise (Fig. 2), thus decreasing the final SNR, and (ii) when the central sphere used to calculate the mean signal covers regions with artifactually high or low signal, for example, ventricles or blood-rich areas. In such cases, the resulting SNR value might be misleading and we suggest verifying the center of mass in the images to be representative of the signal in the image. We also noticed that the alternative Chang method has limitations when it comes to the ratio of noise-to-tissue in the field of view. In particular, different SNR ranges create different circumstances for the ratio of noise-to-tissue for a correct estimation of the standard deviation of noise in the image (Chang et al., 2005). If the ratio is small, SNR-Chang overestimates the true SNR as the quantitative comparison with the low SNR-Standard in line with the visual comparison showed. It should be noted that SNR-Chang, different to SNR-Standard (which uses the b0 images only), creates an average SNR across all diffusion directions except b0, resulting in a less interpretable value for multiple diffusion weightings (i.e., b-values).

To retain the high versatility of AIDAqc, both SNR algorithms were included, and for most cases, the majority voting approach still resulted in a stable detection of low-quality images.

Similar to the SNR calculation, the motion detection was successfully automated in AIDAqc without the need to manually draw regions or perform separate tissue segmentations. Nevertheless, the user should be aware that the mutual information calculation cannot distinguish between animal and hardware motion if both have similar effects, for example, producing a drift in the image stack. An extreme example dataset is*117_m_Ri*with the highest motion severity of all functional datasets. However, in this case, the animals were paralyzed and artificially ventilated, which practically reduces animal motion to a minimum (Bukhari et al., 2017). The high motion severity can be explained by a hardware limitation of the used gradient system in the 11.7T Bruker scanner, creating thermal artifacts at a high-duty cycle, that is, long acquisitions of thin slices with short repetition time.

#### 4.5.3 Validation approaches

In an ideal validation scenario, manual raters would examine every slice, across all time points, repetitions, and diffusion directions, mirroring the extensive analysis performed by AIDAqc. However, such a level of manual scrutiny is impractical and leads to highly variable results as shown in the inter-rater comparison. It underscores the necessity for automated tools and the challenges faced in achieving comprehensive quality control. The full transparency of sharing the code, reporting results, and related data was implemented to ensure future improvements are implemented by the community. In an alternative validation scenario, the tSNR maps or mutual information plots could be provided for the expert raters as an additional level of quality assessment.

Future AIDAqc developments should strike an optimal balance between sensitivity and precision. High sensitivity ensures the tool’s ability to detect the majority of outliers or poor-quality data, which is vital for reliable neuroimaging analysis. Simultaneously, high precision is equally important, as it assures users that the data flagged as outliers are indeed of inferior quality. To further increase the precision, alternative approaches could be explored, that is, feeding the images directly without the calculation of quality features into the machine-learning algorithms. However, such an approach would be time consuming and require high computational power that maybe not every user can afford.

## Supplementary Material

Supplementary Material

## Data Availability

Datasets for testing and validating AIDAqc (Table 1) were collected from 19 international MRI laboratories. These datasets (CC BY-NC-SA 4.0 license) organized according to the scheme outlined inKalantari et al. (2023)can be accessed via:https://doi.gin.g-node.org/10.12751/g-node.q82cjj/. This DOI contains all datasets and results of this publication. Datasets remained unchanged during the revision process, while the*output*and*code*folder contained additional content representing changes during the revision. Access to the live datasets’ content can be facilitated by clicking on the “Browse Repository” button on the GIN DOI, where further details are available. The published data listed inSupplementary Table 2can be obtained through the provided links/references in the table.
