# Supplementary material for: Automated quality control of small animal MR neuroimaging data
Source: Imaging Neurosci (Camb). 2024 Oct 17;2:imag-2-00317. doi: 10.1162/imag_a_00317 (PMC11980538; doi:10.1162/imag_a_00317)
Supplement: Supplementary Material [file imag_a_00317-supp.pdf]

SUPPLEMENTARY FIGURES AND TABLES

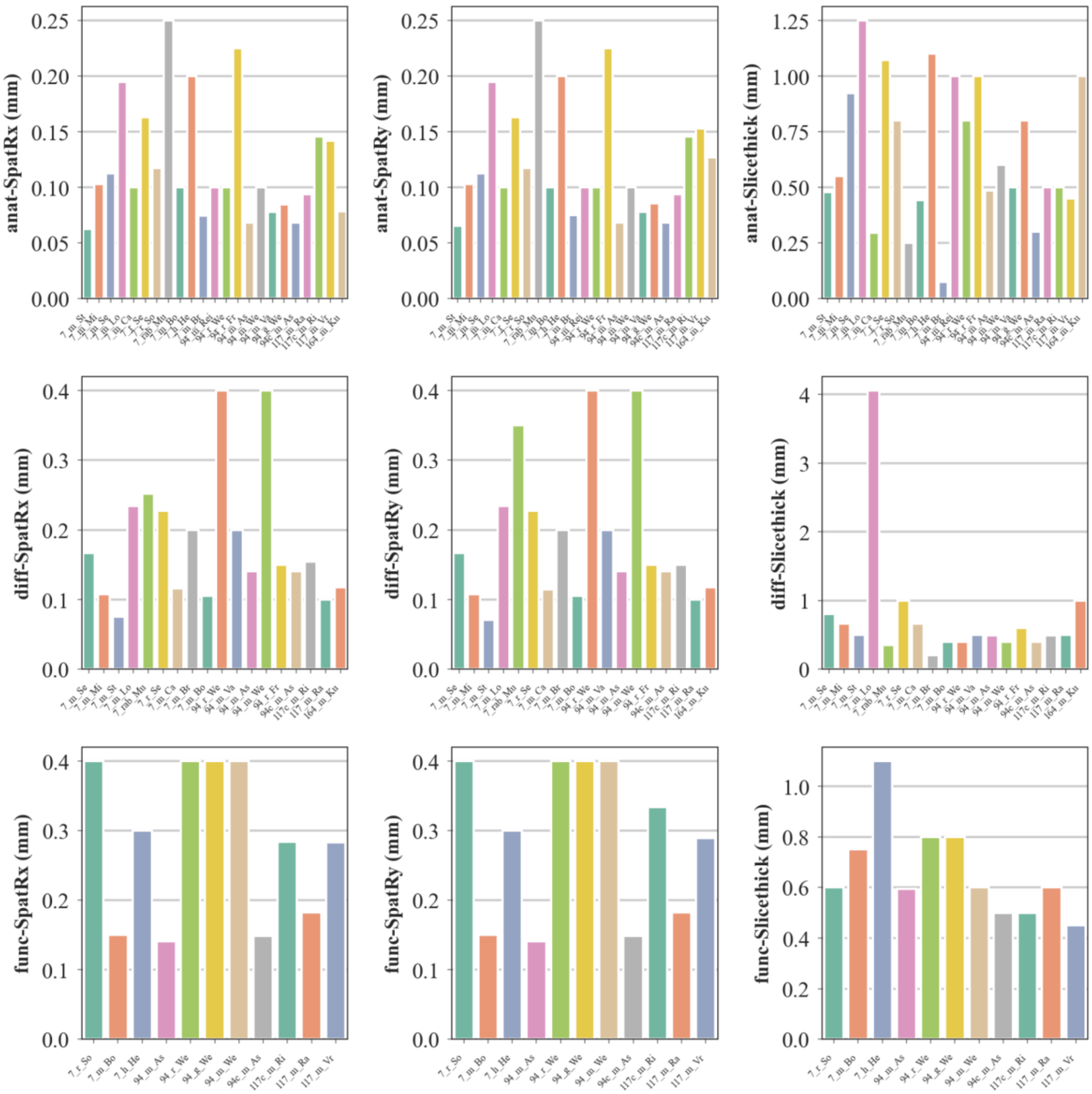

Supplementary Figure 1: Summary of spatial resolution for all studies. Spatial resolution in x- and y-direction (SpatRx, SpatRy) and slice thickness for all studies sorted into anatomical (anat), diffusion (diff), and functional (func) scans.

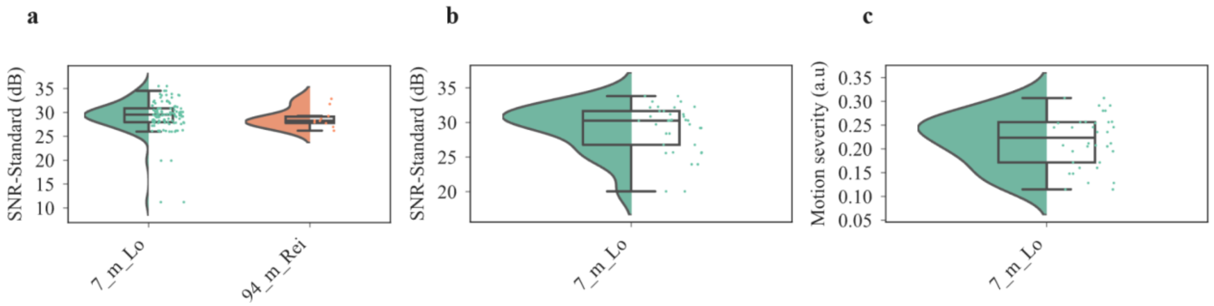

Supplementary Figure 2: Summary of quality features for anatomical scans. (a) SNR Standard for anatomical scans. (b) SNR Standard of diffusion scans. (c) Motion severity values for diffusion scans. This plot is the extension of Fig. 5 in the main manuscript for the two abdominal datasets 7_m_Lo and 94_m_Rei.

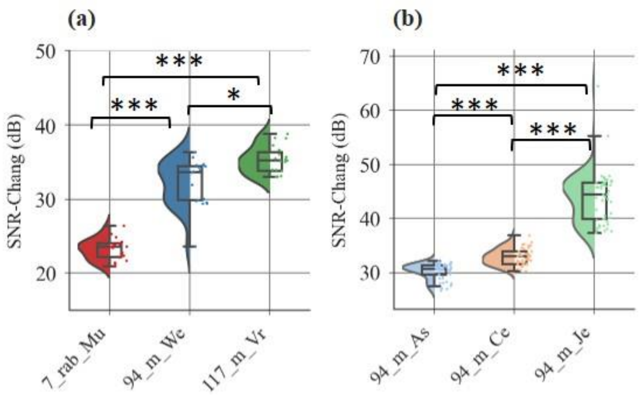

Supplementary Figure 3: Comparison of the field strength and voxel size effect on SNR. Significant differences were observed for SNR values (using the Chang method) between selected datasets with (a) different field strengths and (b) different voxel sizes. Voxel sizes: 7_rab_Mu (0.25x0.25x0.25 mm), 94_m_As (0.068x0.068x0.3 mm), 94_m_Ce (0.078x0.078x0.5), 94_m_Je (0.15x0.15x0.5), 94_m_We (0.1x0.1x0.4 mm), 117_m_Vr (0.142x0.153x0.45 mm). To determine significance, a Kruskal-Wallis test was initially conducted. Subsequently, a post hoc Dunn's multiple comparison test using the Holm–Bonferroni correction for p-value calculation was used. Significant differences between groups are shown as p<0.05 (*), p<0.01 (**), p<0.001 (***).

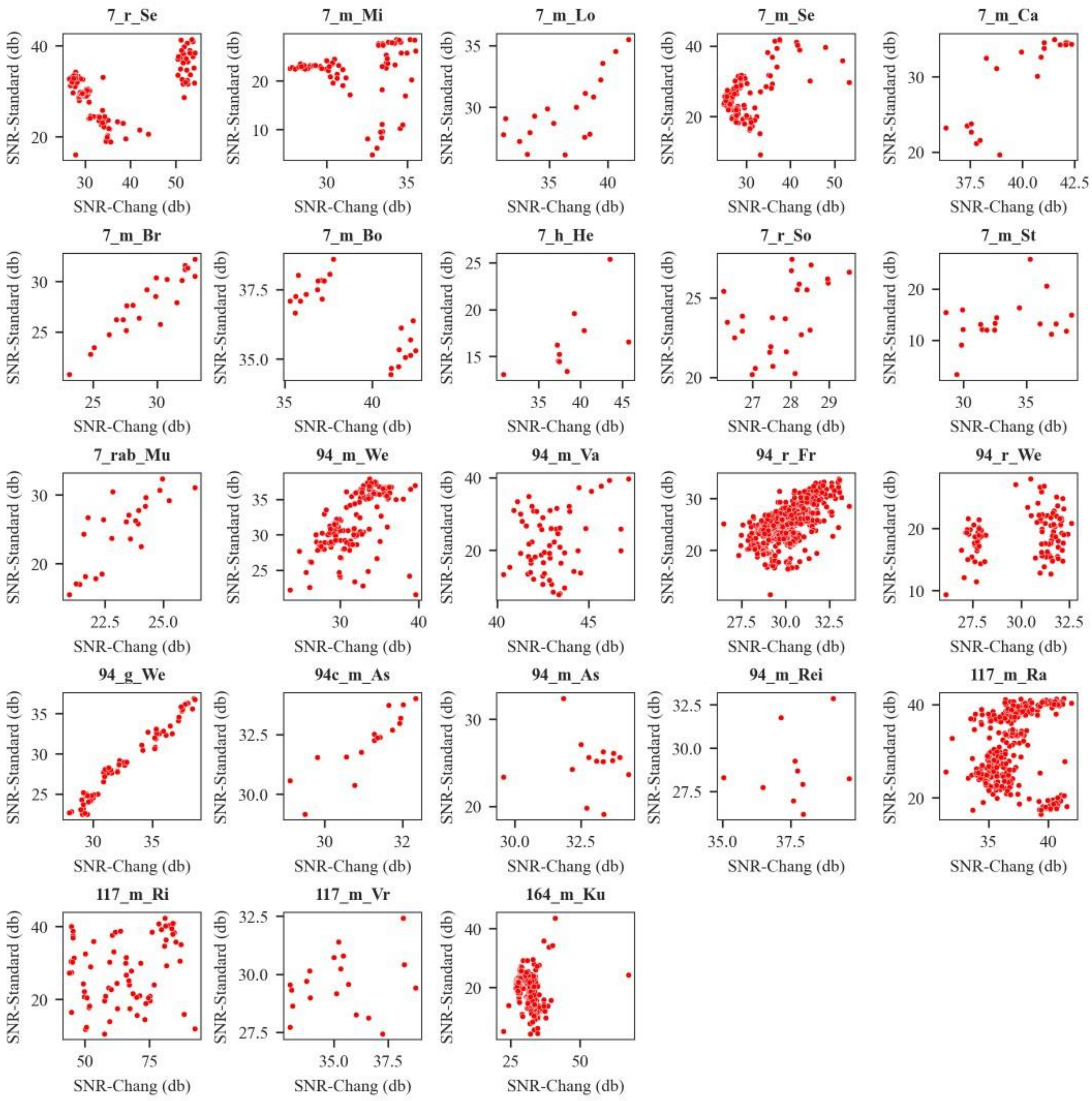

Supplementary Figure 4: Correlation of SNR-Standard and SNR-Chang for all anatomical datasets.

Role of the rating threshold for the agreement between manual raters and AIDAqc

In the following analysis, the predicted truth by manual raters was compared against the ratings generated by AIDAqc. Datasets with no true positive and/or false negative (TP+FN>0) files were excluded to reduce the bias on the classification statistics, i.e., mitigate the impact of datasets where manual raters did not classify any files as “bad quality” (Table I). Further, different thresholds for the number of manual raters and AIDAqc outlier algorithms were compared.

The following metrics were used for a quantitative comparison:

1. Sensitivity: Out of all the actual “bad quality” datasets, how many were identified by AIDAqc as “bad quality” data?
   $Sensitivity = \frac{TP}{TP+FN}$
2. Specificity: Out of all the “no bad quality” datasets, how many were identified by AIDAqc as such?
   $$Specificity = \frac{TN}{TN + FP}$$
3. Accuracy: Out of all the classifications, what was the rate of correct classifications?
   $Accuracy = \frac{TP+TN}{TP+TN+FP+FN}$

In the first comparison, the threshold for the manual voting was varied and the classification was statistically compared against all combinations of AIDAqc thresholds. The overall accuracy (rate of correct classifications) was relatively high across all manual rater voting thresholds for anatomical (0.77 ± 0.21), diffusion (0.70 ± 0.25), and functional (0.65 ± 0.27) scans (Supplementary Fig. 5a). The accuracy reached a maximum for anatomical and functional scans with the manual rater voting threshold set to four and for diffusion scans with the threshold set to five. For specificity, i.e., “how many true negatives” were identified by AIDAqc, the optimal manual votings threshold was four for anatomical (0.95 ± 0.09) and functional (0.93 ± 0.15) scans, respectively, and five for diffusion scans (0.94 ± 0.08) (Supplementary Fig. 5b). Sensitivity, a measure of how many true positive files were identified by AIDAqc, reached a peak with the manual rating

threshold of two for diffusion (0.05 ± 0.13) and functional (0.05 ± 0.14) scans, respectively, and for anatomical sequences with a threshold set to four (0.77 ± 0.21) (Supplementary Fig. 5c).

In the second comparison, the threshold for the AIDAqc voting was varied and the classification was statistically compared against all combinations of manual thresholds. Here, a consistent trend was observed: accuracy and specificity increased with the number of AIDAqc votes, whereas sensitivity reached the maximum already with the AIDAqc voting threshold set to one (Supplementary Fig. 5d-e). Average accuracy overall values for anatomical, diffusion, and functional scans were (0.77±0.21), (0.70±0.25), and (0.65±0.27) respectively. For specificity, these values were (0.95±0.09), (0.94±0.08) and (0.93±0.15). The highest sensitivity values for AIDAqc were reached with a threshold set to one for all sequence types. The average and std across all values for anatomical, diffusion, and functional were (0.05 ± 0.14), (0.05 ± 0.13), and (0.05 ± 0.14) respectively.

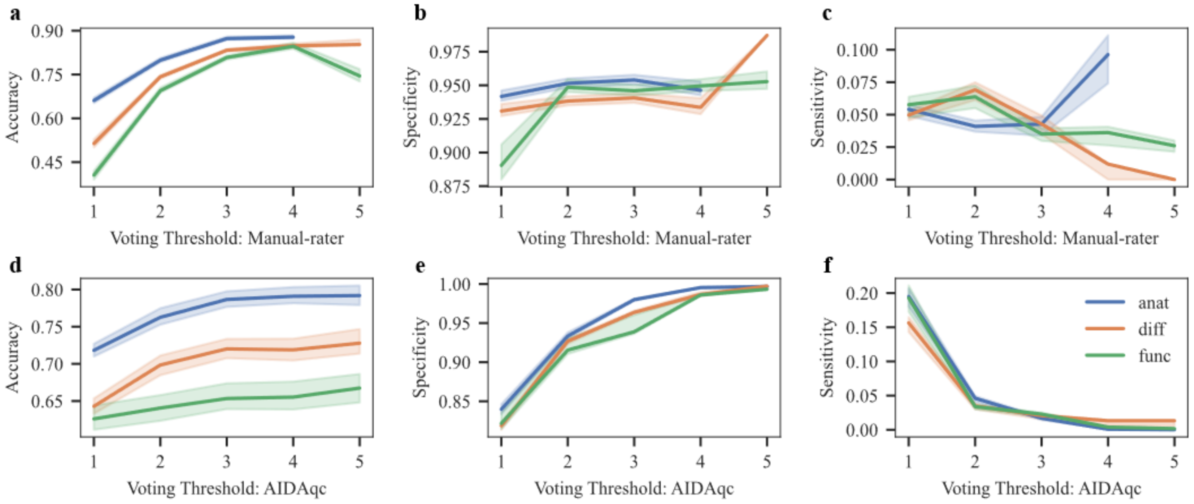

Supplementary Figure 5: Statistical analysis of the classification. Panels (a-c) showcase the dynamic interplay among accuracy, specificity, and sensitivity across varying manual rater voting thresholds. Panels (d-f) offer a parallel examination using AIDAqc voting thresholds. Each color represents a distinct sequence (anat - anatomical, diff - diffusion, and func - functional). The 30% confidence interval encapsulates the statistical uncertainty inherent in the plotted metrics. It is noteworthy that the condition (TP+TN)>0 was applied before extracting these values; thus, some datasets ended up with no bad quality data when the manual-rater threshold was set to 5, as evident in the anatomical sequence in (a, c).

Application of the outlier detection

AIDAqc does not directly measure quantitative post-processing values. However, it is also important to note that the quality assessment based on factors like SNR or motion can indirectly affect processes such as brain extraction as shown in Supplementary Fig. 6, for the case with majority vote = 3. Problems in the subsequent processing steps can consequently lead to less precise quantitative values (Fig. 9).

https://gin.g-node.org/Aswendt_Lab/2023_Kalantari_AIDAqc/src/master/output/SupplementFigures6.pdf

Supplementary Figure 6: Original DWI images and corresponding FA map pairs of all subjects from the dataset 94c_m_As and 94_m_As. The majority vote for the image to be an outlier is written above each DWI image.

https://gin.g-node.org/Aswendt_Lab/2023_Kalantari_AIDAqc/src/master/output/SupplementFigures7.pdf

Supplementary Figure 7: Overview of all subjects and extension of Fig. 10 in the manuscript. For each subject, 4 different slices, and the registered atlas of those 4 different slices are shown. For the subject with the Majority Vote of 5, it can be seen that the brain extraction has completely failed along with the bias filed correction, leading to a faulty atlas registration.

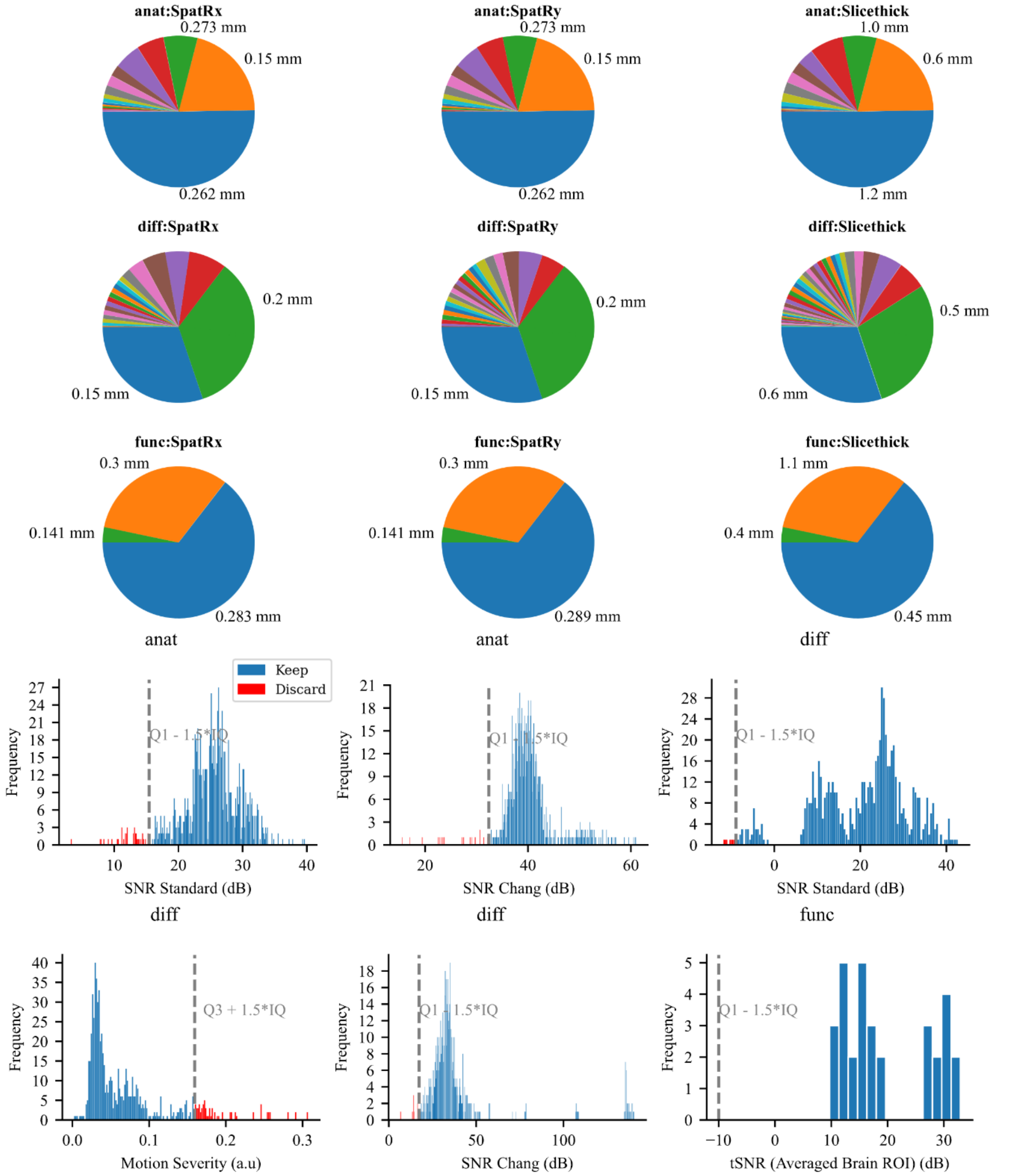

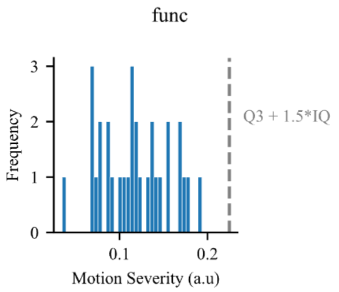

	A	B	C	D	E	F	G	H	I
1	Path	Image	Sequence	One_class_SVM	IsolationForest	LocalOutlierFactor	EllipticEnvelope	statistical_method	Votings
2	\\134.9	anat_V	anat	FALSE	TRUE	FALSE	TRUE	FALSE	2
3	\\134.9	anat_V	anat	FALSE	TRUE	FALSE	TRUE	FALSE	2
4	\\134.9	anat_V	anat	FALSE	TRUE	TRUE	FALSE	FALSE	2
5	\\134.9	anat_V	anat	FALSE	TRUE	FALSE	TRUE	FALSE	2
6	\\134.9	anat_V	anat	FALSE	TRUE	TRUE	FALSE	FALSE	2
7	\\134.9	anat_V	anat	FALSE	TRUE	TRUE	FALSE	FALSE	2
8	\\134.9	anat_V	anat	FALSE	TRUE	FALSE	TRUE	FALSE	2
9	\\134.9	anat_V	anat	FALSE	TRUE	FALSE	TRUE	FALSE	2
10	\\134.9	anat_V	anat	FALSE	TRUE	TRUE	FALSE	FALSE	2
11	\\134.9	diff_D1	diff	FALSE	TRUE	TRUE	FALSE	FALSE	2
12	\\134.9	diff_D1	diff	FALSE	TRUE	TRUE	FALSE	FALSE	2
13	\\134.9	diff_OI	diff	FALSE	TRUE	TRUE	FALSE	FALSE	2
14	\\134.9	diff_OI	diff	FALSE	TRUE	TRUE	FALSE	FALSE	2
15	\\134.9	diff_D1	diff	TRUE	TRUE	FALSE	FALSE	FALSE	2
16	\\134.9	func_fl	func	FALSE	TRUE	FALSE	TRUE	FALSE	2

Supplementary Figure 8: AIDAqc output statistics for all NIfTI datasets (Table I and Supplementary Table I)**.** Pie diagrams show spatial resolution (in mm) for x, y, z direction. Bar plots show distribution of quality metrics. The table shows outliers not considered a statistical outlier but detected by the other algorithms.

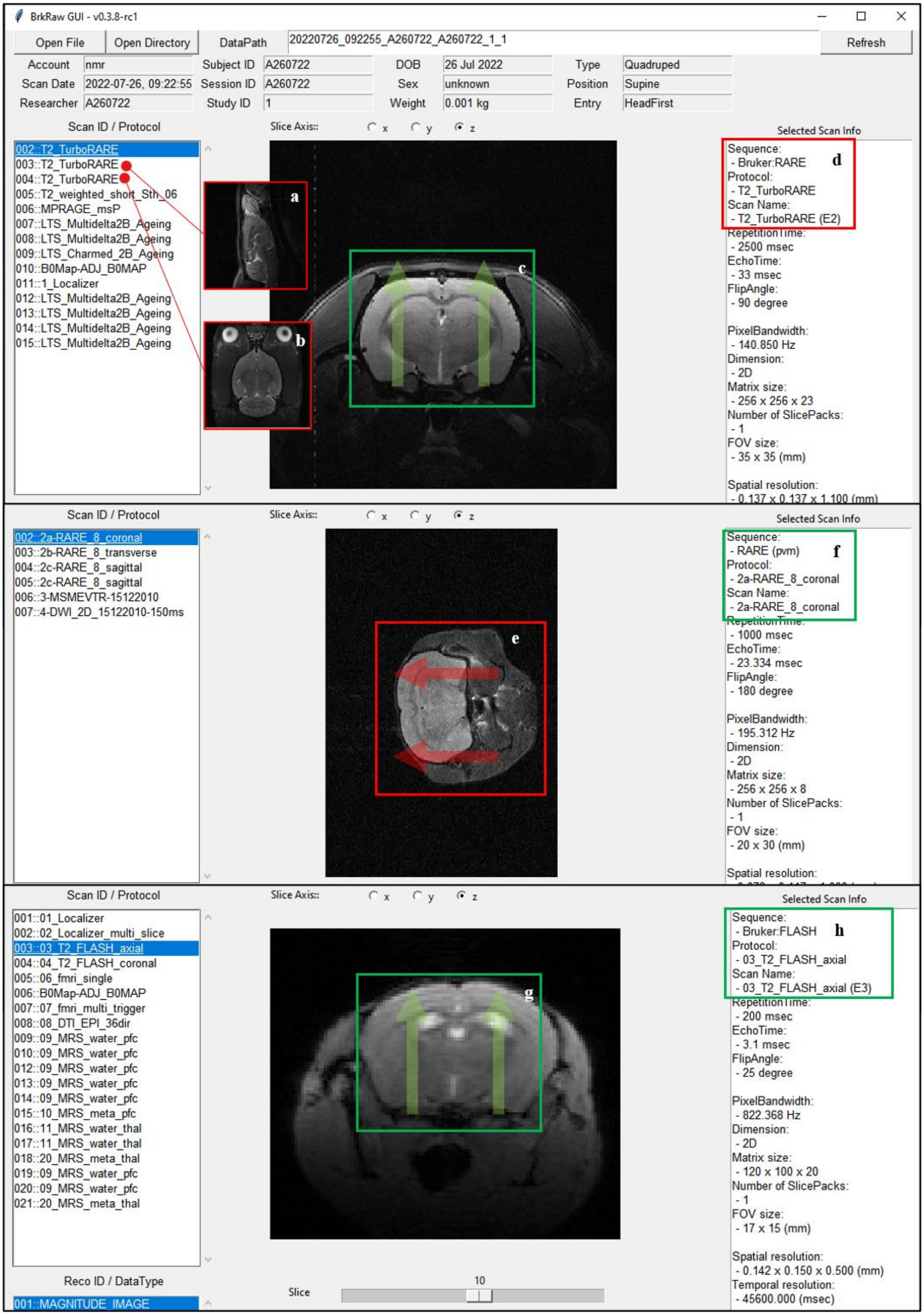
BrkRaw GUI - v0.3.8-rc1
Open File
Open Directory
DataPath
20220726_092255_A260722_A260722_1_1
Refresh
Account
nmr
Scan Date
2022-07-26, 09:22:55
Researcher
A260722
Subject ID
A260722
Session ID
A260722
Study ID
1
DOB
26 Jul 2022
Sex
unknown
Weight
0.001 kg
Type
Quadruped
Position
Supine
Entry
HeadFirst
Scan ID / Protocol
002::T2_TurboRARE
003::T2_TurboRARE
004::T2_TurboRARE
005::T2_weighted_short_Sth_06
006::MPRAGE_msP
007::LTS_Multidelta2B_Ageing
008::LTS_Multidelta2B_Ageing
009::LTS_Charmed_2B_Ageing
010::B0Map-ADJ_B0MAP
011::1_Localizer
012::LTS_Multidelta2B_Ageing
013::LTS_Multidelta2B_Ageing
014::LTS_Multidelta2B_Ageing
015::LTS_Multidelta2B_Ageing
Slice Axis::
x
y
z
a
b
c
Selected Scan Info
Sequence:
- Bruker:RARE
Protocol:
- T2_TurboRARE
Scan Name:
- T2_TurboRARE (E2)
d
RepetitionTime:
- 2500 msec
EchoTime:
- 33 msec
FlipAngle:
- 90 degree
PixelBandwidth:
- 140.850 Hz
Dimension:
- 2D
Matrix size:
- 256 x 256 x 23
Number of SlicePacks:
- 1
FOV size:
- 35 x 35 (mm)
Spatial resolution:
- 0.137 x 0.137 x 1.100 (mm)
Scan ID / Protocol
002::2a-RARE_8_coronal
003::2b-RARE_8_transverse
004::2c-RARE_8_sagittal
005::2c-RARE_8_sagittal
006::3-MSMEVTR-15122010
007::4-DWI_2D_15122010-150ms
Slice Axis::
x
y
z
e
Selected Scan Info
Sequence:
- RARE (pvm)
Protocol:
- 2a-RARE_8_coronal
Scan Name:
- 2a-RARE_8_coronal
f
RepetitionTime:
- 1000 msec
EchoTime:
- 23.334 msec
FlipAngle:
- 180 degree
PixelBandwidth:
- 195.312 Hz
Dimension:
- 2D
Matrix size:
- 256 x 256 x 8
Number of SlicePacks:
- 1
FOV size:
- 20 x 30 (mm)
Spatial resolution:
Scan ID / Protocol
001::01_Localizer
002::02_Localizer_multi_slice
003::03_T2_FLASH_axial
004::04_T2_FLASH_coronal
005::06_fmri_single
006::B0Map-ADJ_B0MAP
007::07_fmri_multi_trigger
008::08_DTI_EPI_36dir
009::09_MRS_water_pfc
010::09_MRS_water_pfc
012::09_MRS_water_pfc
013::09_MRS_water_pfc
014::09_MRS_water_pfc
015::10_MRS_meta_pfc
016::11_MRS_water_thal
017::11_MRS_water_thal
018::20_MRS_meta_thal
019::09_MRS_water_pfc
020::09_MRS_water_pfc
021::20_MRS_meta_thal
Slice Axis::
x
y
z
g
Reco ID / DataType
001::MAGNITUDE_IMAGE
Slice
10
Selected Scan Info
Sequence:
- Bruker:FLASH
Protocol:
- 03_T2_FLASH_axial
Scan Name:
- 03_T2_FLASH_axial (E3)
h
RepetitionTime:
- 200 msec
EchoTime:
- 3.1 msec
FlipAngle:
- 25 degree
PixelBandwidth:
- 822.368 Hz
Dimension:
- 2D
Matrix size:
- 120 x 100 x 20
Number of SlicePacks:
- 1
FOV size:
- 17 x 15 (mm)
Spatial resolution:
- 0.142 x 0.150 x 0.500 (mm)
Temporal resolution:
- 45600.000 (msec)

Supplementary Figure 9: Orientatiation in datasets. Three exemplary subjects from three different datasets from Table I. Multiple TurboRARE sequences with the same sequence, protocol, and scan names (d) but scanned with different orientations (a), (b), wherein the second subject, this was mentioned in the protocol and scan name (f). Subject alignment is supine in (c) whereas in (e) it is set as supine but right to left. In the third subject, both subject alignment and orientation were done in a standard norm (g,h). AIDAqc's current limitation lies in comparing different orientations such as (a), (b), and (c), which may raise questions. It might not lead to outliers if the cohort of subjects has the same sequences, but it still may be questionable and would require further automatic adjustment in terms of how the algorithm extracts the SNR, motion, and ghosting.

Supplementary Table I: Summary of datasets used for developing AIDAqc

	Repository	Datasets	Species	Scanner (Vendor, field strength/bore size, electronics, gradient, Tx/Rx coils)	Modalities	Sequences	Data format	Sample size (n)
**1**	**Aswendt**	**94_m_As**	Mouse brain	Bruker Biospec 94/20 with AVANCE II electronics. Gradient: B-GA12SHP (660 mT/m and slew rates up to 4570 T/m/s). TX/RX: 1H quadrature cryogenic surface coil or 1H mouse brain quadrature surface.	T2w, rs-fMRI, DTI	TurboRARE, GE-EPI, SE-EPI	Bruker raw	32
**2**	**Aswendt**	**94c_m_As**	Mouse brain	Bruker Biospec 94/20 with AVANCE II electronics. Gradient: B-GA12SHP (660 mT/m and slew rates up to 4570 T/m/s). TX/RX: 1H quadrature cryogenic surface coil or 1H mouse brain quadrature surface.	T2w, rs-fMRI, DTI	TurboRARE, EPI_DTI_BIS,GRE_FMRI	Bruker raw	32
**3**	**Boehm-Sturm**	**7_m_Bo**	Mouse brain	Bruker Biospec 70/20USR with AVANCE III HD electronics. Gradient: BGA-12S HP (440 mt/m and slew rates up to 3440 T/m/s). TX/RX: 1H quadrature cryogenic surface coil	T2w, DTI	TurboRARE, DTI_EPI_SEG, T2STAR	Bruker raw	27
**4**	**Carnevale**	**7_m_Ca**	Mouse brain	Bruker Pharmascan 70/16US mm with AVANCE III HD electronics. Gradient: BGA9SHP, Tx: 1H RF circularly polarized 72 mm volume coil. RX: 1H mouse brain quadrature surface.	3D-T2/DTI	TurboRARE, DTI_EPI	Bruker raw	20
**5**	**Franx**	**94_r_Fr**	Rat brain	Varian/Agilent 94/210 mm bore. Gradient: 205_120_HD gradient (400 mT/m), Tx: 80 mm Helmholtz coil, Rx:15 mm inductively coupled surface coil receive	T2w, DWI, T1w	multishot EPI spin-echo, 2D gradient echo	Nifti	722
**6**	**Hekmatyar**	**7_h_He**	Hamster brain	Bruker Biospec 70/20. Gradient: NA. 2x2 1H Rat brain surface array coil 400 mT/mm 12 cm inner diameter)	T2w, rs-fMRI	2D Single shot spin echo EPI (SE-EPI)	Nifti	10
**7**	**Kurniawan**	**164_m_Ku**	Mouse brain	Bruker Biospec 164/89 vertical. Gradient: micro 2.5 gradient max 1.5T/m using Great 60 gradient amplifier. Avance II electronics. Tx/Rx: SAW 20 mm mouse head coil.	T2w, DTI	RARE, DWI	Bruker raw	168
**8**	**Micotti**	**7_m_Mi**	Mouse brain	Bruker Biospec 70/30 with AVANCE III electronics. Gradient: BGA12SL gradient (440 mT/m and slew rates up to 3440 T/m/s). Tx: 1H RF circularly polarized 72 mm volume coil. RX: 1H mouse brain quadrature surface.	T2w, DTI	RARE_POSIZ_AXIAL, DTI_RETE	Bruker raw	21
**9**	**Muñoz-Moreno**	**7_rab_Mu**	Rabbit brain[1]	Bruker Biospec 70/30 with AVANCE III electronics. Gradient: BGA12S (400 mT/m, 12 cm inner diameter) . Tx: NA. Rx: NA.	T1w, DWI	7T T1w (MDEFT),DWI	Nifti	24
**10**	**Brinton**	**7_m_Br**	Mouse brain	Bruker Biospec 70/20 and Bruker 300 1H 2x2 mouse brain surface array or 300 1H 2x2 rat brain surface array	T2w, DWI	TurboRARE, 3D DTI_EPI	Nifti	20

11	**Ramos-Cabrer**	**117_m_Ra**	Mouse brain	Bruker Biospec USR 117/16 with AVANCe III electronics. Gradient: BGA-9S (750 mT/m strength, and 6600 T/m/s slew rate). Tx: Bruker's 500 1H RF circularly polarized 72 mm volume coil, RX: Bruker's RF 500 1H – Mouse Brain SUC surface coil OR Neoos Biotech's mouse brain surface coil.	T2w, DTI, rs-fMRI	TurboRARE, FLASH, EPI	Bruker raw	69
12	**Reichardt**	**94_m_Rei**	Mouse abdominal	Bruker Biospec 94/20 mm with Avance3 electronics. Gradient: B-GA 12S., 680 mT/m, Tx/RX .1H mouse quadrature volume coil (38mm ID)	T2w	T2_TRARE	Bruker raw	10
13	**Rivera-Olvera**	**117_m_Ri**	Mouse brain	Bruker Biospec USR 117/16, B-GA9S (600 mT/m and slew rates up to 6000 T/m/s) ; TX/RX: 1H quadrature cryogenic surface coil.	T2w, DTI, rs-fMRI	DTIEPI, FLASH, EPI	Bruker raw	20
14	**Sta Maria**	**7_m_St**	Mouse brain	7T PET-MR system (MR Solutions Ltd, Guildford, UK), bore size: ~ 24 cm, gradient strength: 600 mT/m, coil: 20 mm inner diameter, 18 mm length, quadrature birdcage	T2w, DTI	FSE, FLASH, SE-EPI	Nifti	17
15	**Selim**	**7_m_Se**	Mouse/rat brain	Bruker Biospec 70/30, Bruker 300 1H 2x2 mouse brain surface array or 300 1H 2x2 rat brain surface array	T2w/T1w/DTI	TurboRARE, WEIGHTED_SHORT, SDS	Bruker raw	20/30
16	**Selim**	**7_r_Se**	Mouse/rat brain	Bruker Biospec 70/30, Bruker 300 1H 2x2 mouse brain surface array or 300 1H 2x2 rat brain surface array	T2w/T1w/DTI	TurboRARE, LTS, T2_WEIGHTED_SHORT	Bruker raw	20/30
17	**Soria**	**7_r_So**	Rat brain	7T Bruker Biospec 70/30 horizontal animal scanner (400 mT/m, 12 cm inner diameter), 1H 2x2 rat brain surface array	T2w, rs-fMRI	7T T2w (RARE),rs-fMRI	Nifti	8 (multiple time points)
18	**Van Leeuwen**	**94_m_Va**	In vivo mouse brain	9.4 T Varian/Agilent, quadrature birdcage with ID 35 mm, 115_60_HD gradient (1000 mT/m)	T2w, DTI	TurboRARE	Nifti	131
19	**Vrooman**	**117_m_Vr**	In vivo mouse brain	Bruker Biospec USR 117/16, B-GA9S (600 mT/m and slew rates up to 6000 T/m/s) ; Mouse with single loop 10 mm diameter Rx surface coil and 089/072 mm Tx volume resonator.	T2w, GE-EPI	TurboRARE /rs-fMRI	Nifti	12 (multiple time points)
20	**Wenk**	**94_m_We**	In vivo mouse/rat/gerbil brain	9.4 Bruker Biospec USR 94/20, B-GA12SHP (660 mT/m and slew rates up to 4570 T/m/s) ; Mouse with Array coil + 112/086 volume resonator; Gerbil with single-loop coil ID15 + 112/086 volume resonator; Rat with 075/40 volume resonator	T2w, rs-fMRI, fMRI, DTI	T2_ANATOMY, DTI_EPI_ISO, T2STAR, AUDISTIM	Bruker raw	46/32/25
21	**Wenk/Goncalves**	**94_r_We**	In vivo mouse/rat/gerbil brain	9.4 Bruker Biospec USR 94/20, B-GA12SHP (660 mT/m and slew rates up to 4570 T/m/s) ; Mouse with Array coil + 112/086 volume resonator; Gerbil with single-loop coil ID15 + 112/086 volume resonator; Rat with 075/40 volume resonator	T2w, rs-fMRI, fMRI, DTI	T2_ANATOMY, DTI_EPI_ISO, RSFMRI_EXPERIMENT	Bruker raw	46/32/25

**22**	**Wenk/Michalek**	**94_g_We**	In vivo mouse/rat/gerbil brain	9.4 Bruker Biospec USR 94/20, B-GA12SHP (660 mT/m and slew rates up to 4570 T/m/s) ; Mouse with Array coil + 112/086 volume resonator; Gerbil with single-loop coil ID15 + 112/086 volume resonator; Rat with 075/40 volume resonator	T2w, rs-fMRI, fMRI, DTI	T2_TURBORARE, NON_ROTATING_EPI	Bruker raw	46/32/25
**23**	**Longo**	**7_m_Lo**	In vivo mouse abdominal	7T MicroImaging Bruker Avance III, 1H 30mm TX/RX body volume coil	T2w, DTI	T2 mic_rare, DtiEpi	Bruker raw	20

[1]ex vivo. Abbreviations: DTI (Diffusion Tensor Imaging), NA (details not available), rs-fMRI (resting-state functional MRI), T2w (T2-weighted MRI), Tx (transmit coil), Rx (Receive coil)

Supplementary Table II: Summary of published datasets used for testing AIDAqc

	Repository	Species /Target	Scanner	Sequences	Data types	Ref.
**1**	**Aswendt**	Mouse	9.4T Bruker, cryocoil	T2-weighted, rs-fMRI	Bruker raw	[63] https://doi.org/10.17617/3.5p
**2**	**Jelescu**	Rat	14T Varian System	T2-weighted, DTI, rs-fMRI	Nifti	https://openneuro.org/datasets/ds003520/versions/1.0.2 https://openneuro.org/datasets/ds004441/versions/1.0.1

Supplementary Table III: Confusion matrix visualizing the statistical definition used to classify the data.

Total	***AIDAqc predicted positive (bad quality data)***	***AIDAqc predicted positive (no bad quality data)***
Manual rater assigned positive (bad quality data)	**True positive (TP)**	**False negative (FN)**
Manual rater assigned negative (no bad quality data)	**False positive (FP)**	**True negative (TN)**
